# Temporal and spatial comparisons of angiosperm diversity between eastern Asia and North America

**DOI:** 10.1093/nsr/nwab199

**Published:** 2021-12-01

**Authors:** Haihua Hu, Jianfei Ye, Bing Liu, Lingfeng Mao, Stephen A Smith, Russell L Barrett, Pamela S Soltis, Douglas E Soltis, Zhiduan Chen, Limin Lu

**Affiliations:** State Key Laboratory of Systematic and Evolutionary Botany, Institute of Botany, Chinese Academy of Sciences, China; University of Chinese Academy of Sciences, China; Sino-Africa Joint Research Center, Chinese Academy of Sciences, China; State Key Laboratory of Systematic and Evolutionary Botany, Institute of Botany, Chinese Academy of Sciences, China; Beijing Botanical Garden, Institute of Botany, Chinese Academy of Sciences, China; State Key Laboratory of Systematic and Evolutionary Botany, Institute of Botany, Chinese Academy of Sciences, China; Sino-Africa Joint Research Center, Chinese Academy of Sciences, China; Co-Innovation Center for Sustainable Forestry in Southern China, College of Biology and the Environment, Nanjing Forestry University, China; Department of Ecology and Evolutionary Biology, University of Michigan, USA; National Herbarium of New South Wales, Royal Botanic Gardens and Domain Trust, Australia; Florida Museum of Natural History, University of Florida, USA; Florida Museum of Natural History, University of Florida, USA; Department of Biology, University of Florida, USA; State Key Laboratory of Systematic and Evolutionary Botany, Institute of Botany, Chinese Academy of Sciences, China; Sino-Africa Joint Research Center, Chinese Academy of Sciences, China; State Key Laboratory of Systematic and Evolutionary Botany, Institute of Botany, Chinese Academy of Sciences, China

Comparative approaches that examine floras with similar environments, but different diversity patterns, provide good opportunities to elucidate how uneven distributions of biodiversity were assembled. Eastern Asia (EA) and North America north of Mexico (NA; see definitions of EA and NA in Fig. S1A) share similar geographical environments with comparable latitude, land area and climate, except that NA has a coastline in the west. The two regions harbor more than 12% of all angiosperm species in the world [1,2] and cover six biodiversity hotspots (Fig. S1A) [3]. However, the number of vascular plant species in EA is at least 1.5 times larger than in NA [1,2]. Detailed comparative study of the floras in EA and NA therefore affords an ideal test case for exploring processes that contribute to this highly uneven distribution of plant diversity.

Much of the earlier work focused on comparing taxonomic diversity between eastern EA and eastern NA. However, recent studies have suggested phylogenetic diversity, which incorporates both taxonomic diversity and evolutionary histories of taxa, to be a better measure of biodiversity than only counting and comparing species number [4]. One recent study detected significantly higher species richness and phylogenetic diversity in eastern EA than eastern NA [5]. However, few studies have assessed the phylogenetic diversity patterns between the floras of EA and NA, involving both their eastern and western areas (Fig. S1A). Western parts of EA and NA experienced substantial changes in topography and climate during the Cenozoic, such as the uplift of the Qinghai-Tibetan Plateau (QTP) and the formation of the Rocky Mountains, respectively, which have given rise to high plant diversity in these areas [6–8]. Therefore, a broader comparison of floristic diversity also including western parts of EA and NA is desirable and may shed new light on how the two floras were assembled.

China and the United States of America (USA) cover almost half of the land areas of EA and NA (Fig. S1B), respectively, and harbor much of the plant diversity in the two regions. We herein explore the floristic differences between EA and NA using the well-studied floras of China and the 48 contiguous states of the USA as exemplars, by integrating a newly generated dated phylogeny covering ∼90% of the angiosperm genera of the two countries (93.8% and 88.3% of native genera of China and the USA, respectively), complete species-level trees (including all 41 410 named angiosperm species from the two countries) and comprehensive spatial distribution data. We calculate generic richness and phylogenetic diversity patterns of 1749 grid cells (each of 100 km × 100 km) from China and the USA within a robust dated phylogenetic framework (Figs 1A and S2–4, and Table S1) and aim to: (i) compare angiosperm diversity and divergence patterns between China and the USA; (ii) test heterogeneity in temporal and spatial diversity between eastern and western parts of China and the USA; and (iii) locate areas of conservation priority in both China and the USA by detecting phylogenetic diversity hotspots.

**Figure 1.**
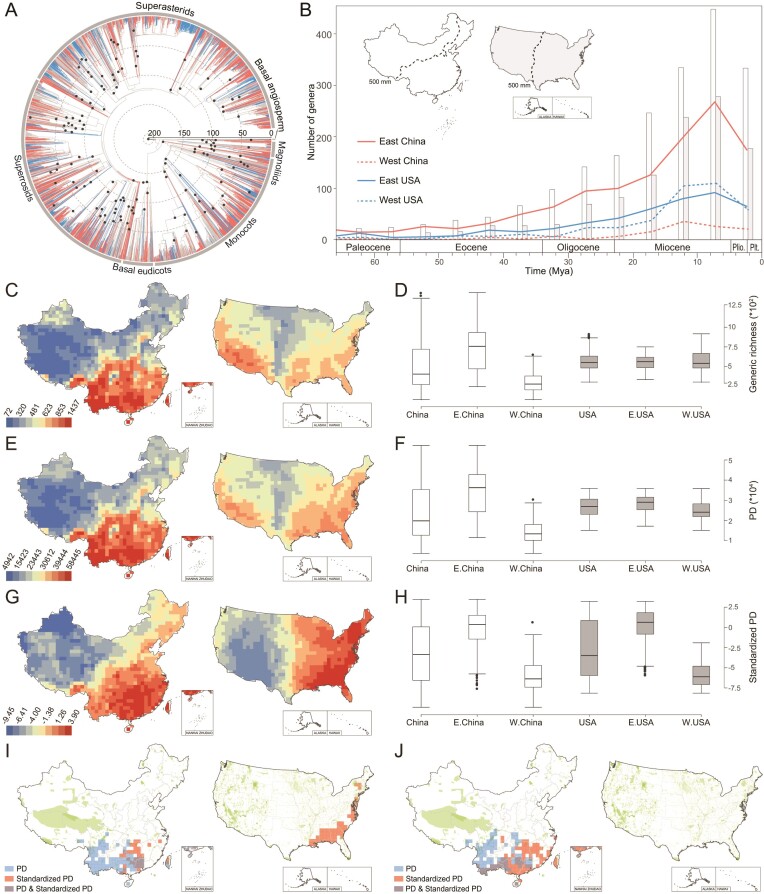
General comparisons of angiosperm diversity between China and the USA, as well as our proposed conservation priorities for the two regions. (A) A dated phylogeny of the angiosperm floras of China and the USA based on a matrix of four plastid genes (*atpB*, *matK*, *ndhF* and *rbcL*) and one mitochondrial gene (*matR*), including 9035 species from 3762 genera and 282 families native to China and the USA. The phylogeny was generated using maximum likelihood (ML), and the time tree was estimated in treePL with 139 calibration points. Major clades/grades, including the basal angiosperm grade, magnoliids, monocots, basal eudicot grade, superrosids and superasterids, are indicated with gray bars outside the circle. Red and blue branches represent genera that occur only in China or the USA, respectively, while genera shared by the two regions are indicated with gray branches. Calibration points are shown with black circles on the nodes (see detailed information in Table S1). (B) Number of angiosperm genera that originated during each 5-million-year period in China (hollow bar), the USA (gray bar), and their eastern and western parts that are divided by the 500-mm isoline of annual precipitation (dashed lines in the maps). Plio.—Pliocene epoch, Plt.—Pleistocene epoch. (C–H) Comparisons of diversity patterns between China and the USA and their eastern and western parts. Geographic patterns of (C) generic richness, (E) phylogenetic diversity (PD) and (G) standardized PD in China and the USA. The values on the colored bar legends represent (C) generic richness, (E) PD and (G) standardized PD values of each grid cell. Boxplots of (D) generic richness, (F) PD and (H) standardized PD in China, the USA and their eastern and western parts divided by the 500-mm isoline of annual precipitation. E. China—eastern China, W. China—western China, E. USA—the eastern USA, W. USA—the western USA; median—solid line in the box, box—interquartile range (25% and 75%), whiskers—5% and 95% intervals. (I,J) Conservation priorities identified based on grid cells with the top 5% PD (blue) and top 5% standardized PD (red) in China and the USA at the (I) genus and (J) species levels, with protected land areas highlighted in green on the maps. The top 5% PD and the top 5% standardized PD are defined as the top 5% grid cells with the highest PD and standardized PD values over the two regions. Grid cells with both the top 5% PD and the top 5% standardized PD are indicated in gray. Maps of nature reserves are adapted from the World Database on Protected Areas (WDPA) (https://www.protectedplanet.net/). Review drawing number for maps: GS(2021)7893.

## GENERAL COMPARISONS OF ANGIOSPERM DIVERSITY

Our study reveals that China possesses higher richness (2858 vs. 1992 genera) of angiosperm genera than the USA. Focusing on eastern parts of China and the USA, previous work demonstrated that eastern China has higher phylogenetic diversity than the eastern USA [5]. With western parts of the two regions included, our study suggests that China also possesses higher Faith's phylogenetic diversity (PD, see Supplementary Methods) of angiosperm genera than the USA (measured in units of time, 90 698 vs. 63 998 million years, Myr). Most lineages (46 of 58 clades recognized as orders) in the angiosperm tree of life have contributed to the PD anomaly favoring China, with only four orders (Boraginales, Canellales, Ericales and Picramniales) having obvious higher PD (PD difference >100 Myr) in the USA (Fig. S3 and Table S2). Moreover, we find that the diversity anomaly is consistent between PD and richness for most lineages, while exceptions are detected in a few lineages. For instance, Boraginales have higher generic richness in China but higher PD in the USA (Fig. S3 and Table S2); the difference in generic richness may reflect biological diversity, different taxonomic concepts between the two countries, or both.

Investigation of temporal divergence patterns reveals that China has more angiosperm genera that originated during each geological timespan than the USA (Fig. 1B) and has also preserved a higher proportion of genera that originated before the Miocene than the USA (29.9% vs. 23.2%, Table 1). This may, at least in part, be explained by both the complex topography in China that provided numerous refugia for ancient lineages and the inference that Chinese flora was less affected by extinction during climate cooling and glaciation [9]. We highlight that southern China bears the signature of both old and new diversification, and is a diversity center for both anciently and recently originated genera (Fig. S5). This finding is congruent with a previous study, which found that southern China tends to be both a ‘museum’ and a ‘cradle’ for angiosperm genera [7]. In the USA, genera of pre-Miocene origin are mainly distributed in the southeastern coastal areas with only a few in the southwestern USA (Fig. S5A), while genera of post-Miocene origin mostly occupy the southwest (Fig. S5B). Indeed, the eastern USA is dominated by deciduous forest, while the western USA mainly comprises more recently assembled floras (e.g. prairie, steppe, montane and desert biomes), except for the relatively old Mediterranean flora in coastal regions [8].

**Table 1.** Number and percentage of angiosperm genera that may have originated during each geological timespan in China and the USA.

Geologic timespan	Number of genera in China (%)	Number of genera in the USA (%)
Jurassic	2 (0.1%)	1 (0.1%)
Cretaceous	95 (4.7%)	27 (2.5%)
Paleocene	47 (2.4%)	18 (1.7%)
Eocene	195 (9.7%)	82 (7.6%)
Oligocene	261 (13.0%)	122 (11.3%)
Miocene	1053 (52.5%)	644 (59.7%)
Pliocene	221 (11.0%)	115 (10.7%)
Pleistocene	130 (6.5%)	70 (6.5%)

## STRIKING EAST-WEST HETEROGENEITY IN DIVERGENCE TIMES AND DIVERSITY

Our study reveals that China shows stronger east-west heterogeneity in diversity than the USA across both temporal and spatial scales (both China and the USA were divided into east and west by the 500-mm isoline of annual precipitation, see Supplementary Methods for details). Eastern China has more genera that originated during each geological timespan and shows higher richness and PD than either western China or the USA, but western China has fewer genera that originated during each geological time interval and lower richness and PD than the USA (Figs 1B–F and S6B). Within China, most major lineages of angiosperms also generally have PD centers in eastern China, with only Brassicales, Boraginales and Caryophyllales having centers of PD in western China (Fig. S7).

The pattern of divergence times within the USA is more complex than within China; the east has more genera that originated during the Paleogene than the west, but the west then surpassed the east after the middle Miocene (Figs 1B and S6B). The USA also shows inconsistent spatial diversity patterns between the east and west for different measures, with generic richness relatively higher (Fig. 1C–D), but PD, standardized PD and relative PD lower in the west (Figs 1E–H and S8A). Such differences between richness and phylogenetic diversity patterns suggest that the eastern USA has preserved more ancient lineages (with longer branches), while the western USA hosts more young lineages (with shorter branches). Our spatial phylogenetic analyses at the species level also detected a striking east-west difference in the distribution of branch lengths, a finding that supports the eastern USA having more ancient lineages (Fig. S9), congruent with the results of Mishler *et al.* (2020) [8].

Although China shows greater east-west deviation in divergence times and diversity than the USA, the two floras share the signature of an older east and a younger west. Heterogeneity in phylogenetic diversity between eastern and western China and the potential driving forces have been well documented by previous studies [7]. Our study again highlights the striking east-west difference in evolutionary histories for the flora of the USA. Although the USA was impacted significantly by glacial oscillations during the Quaternary [9], refugia persisted both south of the glaciers and in pockets north of some of the ice sheets in the eastern USA [10]. Persistent refugia during glacial cycles may have protected diverse lineages in the eastern USA, including lineages of ancient origin. In the western USA, our divergence analyses detected a rapid diversification of the angiosperm flora since the middle Miocene (Figs 1B and S6B) and a concentration of lineages with short branches (Figs 1G–H and S8A), for herbaceous genera in particular (Figs S6B, S8B and S10). Independent phylogenetic studies revealed that some herbaceous lineages from the western USA have experienced rapid diversification since the middle Miocene, perhaps in response to the recent orogeny and an increasingly arid climate in these areas (Fig. S6A and Table S3) [6].

## PHYLOGENETIC DIVERSITY HOTSPOTS AND CONSERVATION PRIORITIES

An increasing number of studies indicate that conservation efforts should focus on the protection of regions with high phylogenetic diversity, rather than species richness, to better prepare for uncertain environmental changes in the future [4]. This assessment of phylogenetic diversity for Chinese and USA floras allows us to make conservation recommendations across portions of two continents (Figs 1I–J and S11). The eastern USA has the top 5% standardized PD when all genera in China and the USA are treated as a single sampling pool (Fig. 1I), and the hotspots are all part of the North American Coastal Plain biodiversity hotspot [3]. However, protection zones in the eastern USA are fragmented, largely due to human activities, while the western USA is covered by relatively large areas with some measure of protection (Fig. 1I). Similarly, southern China has areas with both the highest 5% PD and the highest 5% standardized PD, in agreement with Lu *et al.* (2018) [7], but is only covered by fragmented protected nature reserves (Fig. 1I). Notably, phylogenetic diversity hotspots detected in this study also possess higher phylogenetic endemism (Fig. S12). Our study thus suggests that more conservation areas are needed that closely correspond to areas of high phylogenetic diversity in order to improve plant conservation efforts in southern China and the eastern USA. This type of approach will better preserve lineages with greater genetic diversity and unique physiological adaptations that are potentially beneficial for ecosystem functioning and human health.

The species-level analyses did not detect any phylogenetic diversity hotspots in the eastern USA (Fig. 1J). This result may not be surprising given that genera (and larger clades) represent larger units of genetic diversity and deeper cladogenesis in the tree of life compared with species and may thus represent different patterns of phylogenetic diversity, indicating different histories and processes of the two floras in relatively recent times.

Our current study provides novel insights into the assembly of disjunct floras across much of the northern hemisphere, a topic that has been of interest to biologists for more than 250 years [5,9]. In particular, our phylogenetic diversity analyses support the recognition of the 36th biodiversity hotspot, the North American Coastal Plain [3]. We show that phylogenetic diversity is a key metric in biodiversity conservation. It can be used to identify geo-regions with maximum underlying diversity (e.g. the extent of genetic diversity, evolutionary history or functional diversity of a flora), and is of great potential value to policy makers and land managers. Although a growing number of studies emphasize the importance of applying phylogenetic diversity in conservation planning [4], most conservation strategies are still largely based on taxonomic richness (such as richness of all species, rare species and endemic species). There is still a great deal to learn about the importance of phylogenetic diversity and its implications for biodiversity conservation at both a local and global scale.

## Supplementary Material

nwab199_Supplemental_File
