# Supplementary material for: Temporal and spatial comparisons of angiosperm diversity between eastern Asia and North America
Source: Natl Sci Rev. 2021 Dec 1;9(6):nwab199. doi: 10.1093/nsr/nwab199 (PMC9271013; doi:10.1093/nsr/nwab199)
Supplement: nwab199_Supplemental_File [file nwab199_supplemental_file.docx]

**SUPPLEMENTARY INFORMATION**

**Temporal and spatial comparisons of angiosperm diversity between eastern Asia and North America**

**Authors:** Haihua Hu^1,2,3^, Jianfei Ye^1,4^, Bing Liu^1,3^, Lingfeng Mao^5^, Stephen A. Smith^6^, Russell L. Barrett^7^, Pamela S. Soltis^8^, Douglas E. Soltis^8,9^, Zhiduan Chen^1,3^ and Limin Lu^1,*^

^1^State Key Laboratory of Systematic and Evolutionary Botany, Institute of Botany, Chinese Academy of Sciences, Beijing 100093, China; ^2^University of Chinese Academy of Sciences, Beijing 100049, China; ^3^Sino-Africa Joint Research Center, Chinese Academy of Sciences, Wuhan 430074, China; ^4^Beijing Botanical Garden, Institute of Botany, Chinese Academy of Sciences, Beijing 100093, China; ^5^Co-Innovation Center for Sustainable Forestry in Southern China, College of Biology and the Environment, Nanjing Forestry University, Nanjing 210037, China; ^6^Department of Ecology and Evolutionary Biology, University of Michigan, Ann Arbor, MI 48109, USA; ^7^National Herbarium of New South Wales, Royal Botanic Gardens and Domain Trust, Sydney 2000, New South Wales, Australia; ^8^Florida Museum of Natural History, University of Florida, Gainesville, Florida 32611-7800, USA; ^9^Department of Biology, University of Florida, Gainesville, Florida 32611-8525, USA

^*^**Corresponding Author.** E-mail: [liminlu@ibcas.ac.cn](mailto:liminlu@ibcas.ac.cn)

**This document contains:**

Supplementary Methods 1

Supplementary Results 8

Limitations and caveats 12

Supplementary Figures 13

Supplementary Tables 28

Supplementary References 38

**Supplementary Methods**

***Study area***

In this study, we used the land areas of China and the 48 contiguous states of the USA to represent EA and NA, respectively. China and the USA cover almost half of the land areas of EA and NA (Fig. S1), respectively, and harbor much of the plant diversity in the two regions. Both regions are adjacent to other areas of high species turnover and endemism (EA with southeastern Asia and NA with Mexico), so these boundaries represent pragmatic regions for which abundant occurrence records are available in a consistent format within each country, and genetic data are available for many species. China and the USA therefore represent a useful proxy for comparison of plant diversity between EA and NA [1].

***Floristic data***

We collected county-level distribution data for angiosperms from 34 provinces or equivalent administrative regions in China following Lu *et al.* (2018) [2] and from the 48 contiguous states in the USA using the Biota of North America Program (BONAP) [3] (Fig. S1B). Distribution data in Lu *et al.* (2018) [2] were assembled from nearly all published national and provincial floras, as well as some local floras, checklists, and herbarium records in China. Distribution data in BONAP [3] were assembled from journals and periodicals, Natural Heritage Programs, specimen voucher documentation, and additional reference sources in the USA, including state floras, checklists, and databases.

To minimize potential influence from differences in taxonomic concepts between botanists of China and the USA, species names were standardized according to The Plant List (<http://www.theplantlist.org/>), the names of which were mostly treated based on worldwide-scale taxonomic studies with a consistent approach. For species not included in The Plant List, we checked their names in regional floras and the scientific literature. Infraspecific taxa were combined, and non-native species in each region were excluded. Native versus non-native status was assembled from *Flora of China* [4] and Lu *et al.* (2018) [2] for China and from BONAP, the US Department of Agriculture’s Plants Database (<https://plants.usda.gov/java/>), and the *Flora of North America* [5] for the USA. Considering that species with different growth form respond to environmental variation differently and may differ in their evolutionary histories [6,7], we conducted analyses for woody and herbaceous species separately. Woody plants include trees, shrubs, and subshrubs. Growth form information for each species was assembled from the same data sources as for native status.

We divided the maps of China and the USA into 100 km × 100 km grid cells under equal-area projection using ArcGIS 10.2 (<http://www.esri.com/>), to minimize sampling bias due to the unequal areas of individual counties (the average county size of the two regions approximates 33.4 km^2^). We chose the resolution of 100 km ×100 km following previous large-scale biodiversity analyses [2,8,9]. When a grid cell covers or intersects with a county, the species in the county are assumed to be distributed in that grid cell. To minimize the influence of unequal sampling area, grid cells covering land areas less than 5000 km^2^ were excluded following previous studies [2,10], leaving a total of 1749 grid cells (946 for China and 803 for the USA). The excluded grid cells are all distributed along the borders of the USA and China and cover only 2.4% of the total land area of the two regions. During this process, 283 grid cells <5000 km^2^ that contained 73 species and 10 genera (0.2% of species and 0.2% of genera in China and the USA together) were excluded. The final distribution data contained 2 913 741 records of species-grid cell mappings (1 418 031 records for China and 1 495 710 records for the USA) for 41 410 angiosperm species, representing 4169 genera from 286 families (26 938 species, 2858 genera, and 252 families for China and 14 937 species, 1992 genera, and 205 families for the USA); circumscription of families follows APG IV [11], including 1301 woody genera (987 for China, 458 for the USA), 2700 herbaceous genera (1751 for China, 1422 for the USA), and 168 mixed genera (genera that include both woody and herbaceous species; 120 for China, 112 for the USA), with 465 species, 681 genera, and 170 families shared between China and the USA.

*Phylogeny reconstruction*

We newly generated a phylogeny of angiosperms from China and the USA to conduct analyses within the same evolutionary framework. Sequences of four plastid genes (*atpB*, *matK*, *ndhF*, and *rbcL*) and one mitochondrial gene (*matR*) were used to reconstruct the angiosperm phylogeny of China and the USA. The data sets included sequences from Chen *et al.* (2016) [12] and data newly downloaded from GenBank (<http://www.ncbi.nlm.nih.gov/genbank/>, prior to July, 2017). All sequences for genera native to China and the USA available in GenBank were downloaded using the ‘rentrez’ package in R 3.2.4 [13,14]. We ensured that 10% of the species in each genus were sampled to represent intrageneric diversity; for genera with 1–20 species, 1–3 species were selected. We took infrageneric circumscriptions (subgenus and/or section) into consideration and gave priority to species with more targeted DNA sequences available in GenBank when choosing representative species of each genus. *Amborella trichopoda* Baill., the sister to all other living angiosperms [15,16] and endemic to New Caledonia, was selected as the outgroup. The taxonomic accuracy of sequences was checked against APG IV [11] for interfamilial relationships or recently published, well-supported molecular phylogenetic results for infrafamilial relationships by conducting preliminary maximum likelihood (ML) analyses. If species with unreasonable placement were identified, these sequences were replaced or manually removed. These procedures were repeated until no unexpected lineage positions were detected.

We aligned the sequence matrix for each locus in three steps: first, we aligned a matrix for each gene using MAFFT v.7.305 [17]; second, the alignment of each gene was manually adjusted in BioEdit v.7.2.5 [18]; and third, we re-sorted the sequences according to their phylogenetic positions and manually adjusted the alignments for subsets of closely related species and readjusted the entire data set as necessary. The final matrix for each gene included 2449 (*atpB*), 7625 (*matK*), 3822 (*ndhF*), 7402 (*rbcL*), and 864 (*matR*) sequences. We also generated a phylogenetic tree using *matK* and *rbcL* with less missing sequence data to test whether missing genetic data affect patterns of phylogenetic diversity. The phylogenetic diversity patterns based on the tree reconstructed from *matK* and *rbcL* are congruent with those retrieved from the five-gene tree (*r* > 0.99, *P* < 0.001), suggesting that our results are robust to the higher proportions of missing data in the other three genes. As the phylogeny built on just two genes has lower sampling density and poorer resolution compared with that using five genes, we used the phylogeny based on five genes for downstream analyses. The concatenated matrix of five genes consisted of 9035 species, representing 3762 genera native to China and the USA from 282 families (90.3% of all angiosperm genera found in China and the USA, of which 2682 genera occur in China and 1760 occur in the USA, covering 93.8% and 88.3% of native genera in each region). A partitioned (based on the five gene regions) ML analysis was conducted for the final concatenated data set using RAxML v.8.0.22 [19]. The optimal ML tree was calculated under the GTR + GAMMA model with 1000 bootstrap replicates. The GTR model was used for the transition rates and base frequencies, and rate heterogeneity of sites was modeled with a discretized gamma distribution [20].

*Divergence time estimation and divergence patterns*

We scaled the best ML tree using penalized likelihood (PL) as implemented in treePL (<https://github.com/blackrim/treePL>) [21]. A set of 139 calibrations was selected for the dating analyses (Table S1). The calibration points included carefully vetted fossils (128 of 139) and reliable secondary calibrations (11 of 139); most of the fossils (at least 95.3%) we used have been applied in previous divergence time analyses [22,23] (Table S1). Divergence time estimation procedures then followed Lu *et al.* (2018) [2], with optimal parameters determined by prime analysis as opt = 1, optad = 1, optcvad = 1, and smooth = 1000. Confidence intervals for age estimates were calculated from 100 bootstrap replicates following Magallón *et al.* (2015) [22] and Lu *et al.* (2018) [2]. Bootstrap replicates were generated using RAxML v.8.0.22 [19].

To evaluate the reliability of the estimated divergence times in our study, correlation analyses were conducted to compare our estimated divergence times with those of recent global-scale angiosperm time trees [22-24], of which Smith and Brown (2018) [24] has the largest and most up-to-date time tree for seed plants with 79 881 species. The stem ages of families and genera shared between these studies were extracted for Spearman’s rank correlation analyses in R. We further evaluated the robustness of our divergence time estimation by calculating the proportion of genera with an age difference in each 10-million-year timespan between our study and Smith and Brown (2018) [24].

Proportions of genera that originated before the Miocene and after the Miocene were calculated to identify divergence signatures for floras in China and the USA. To compare the divergence patterns of lineages during the evolutionary history of angiosperms between the two regions, the number of genera that originated during each five-million-year period was estimated for each region. Previous study has uncovered a distinct split in diversity patterns between eastern and western China (1:0.84) by dividing China into two regions along the 500-mm isoline of annual precipitation [2]. We herein also divided the USA into east and west (1:1.12) using the 500-mm isoline of annual precipitation as a boundary, which generally parallels the long recognition of a midcontinental biogeographic break [25]. The two regions both generally have a dry west and humid east, with the dividing lines following annual precipitation, ranging from 12 to 4589 mm in China and 46 to 3345 mm in the USA. We then calculated the number of genera that originated during each five-million-year timespan in the eastern and western parts of both countries. In order to compare divergence patterns between growth forms, the analyses were also conducted for herbaceous and woody genera separately.

*Spatial patterns of diversity and conservation priorities*

Taxonomic richness and Faith’s phylogenetic diversity (PD) [26] were calculated based on our chronogram using the “picante” package in R [27]. PD was calculated as the sum of branches connecting all species in a grid cell. The total PD of a region was calculated with the taxa present in the corresponding region, with lineages not in the region removed from the phylogeny. As Faith’s PD consistently increases with species richness [28,29], a standardized PD was calculated to exclude the accumulative effect of taxonomic richness on the sum of PD using the “picante” package in R [27]. We excluded grid cells containing fewer than 10 genera to avoid underlying distortion of results for standardized PD [30-32]. As a result, 47 grid cells (covering 2.4% of the total area of the two regions) in western China were excluded from the analyses of woody genera alone. Taxa equal to the number of genera in a grid cell were drawn randomly (999 times) across the tree for each grid cell to obtain a mean PD (PD_random_) and a standard deviation of PD_random_ (sd(PD_random_)); standardized PD was then calculated as: (PD_observed_ – PD_random_)/sd(PD_random_), where PD_observed_ was the observed PD for the grid cell. Relative phylogenetic diversity (RPD), which compares PD of the original tree with PD of a comparison tree having the same topology but with all branch lengths equal, was also calculated to identify and distinguish areas of concentration of long branches and short branches following Mishler *et al*. (2014) [33]. To investigate whether PD calculation is affected by unequal sampling density between the two regions, we reanalyzed the phylogenetic diversity patterns with the same proportion of genera sampled between China and the USA (i.e. 88.3%) by randomly dropping 158 genera that occurred in China from the phylogeny (this was repeated three times). The results show that China consistently has higher PD than the USA (mean PD, 87 143 vs. 63 998 million years, Myr). The phylogenetic diversity patterns strongly agree with those we originally retrieved (*r* > 0.99, *P* < 0.001 for both PD and standardized PD), which suggests that our results are robust to the difference in sampling density (5.5%) between the two regions.

We extended analyses of phylogenetic diversity patterns to evaluate hotspots at the species level. We generated complete-species phylogenetic trees by inserting species without sequence data into a backbone tree primarily from Smith and Brown (2018) [24] according to available taxonomic information, using the R package “V.PhyloMaker” [34]. We used “scenario 2” in Jin and Qian (2019) [34] to incorporate branch lengths of the inserted taxa. For 22 401 unsampled species with congeneric species in the backbone tree, the new tip was inserted to a randomly selected node at and below the genus-level basal node; for 1548 unsampled species without congeneric species in the backbone tree, the new tip was randomly inserted to a node in the corresponding family (tips from the same genus were bound to the same node). Finally, a total of 23 949 species and 584 genera native to China and the USA were inserted into the backbone tree, and a complete, 41 410-species phylogenetic tree was assembled after excluding species not native to China and the USA [2,3]. To account for phylogenetic uncertainty caused by randomization of “scenario 2” in Jin and Qian (2019) [34], we generated 10 complete species trees using this method and analyzed the phylogenetic diversity patterns respectively. Correlation analyses of PD and standardized PD values in each grid cell retrieved from the 10 independent species trees were conducted to assess how phylogenetic uncertainty impacts the phylogenetic diversity patterns. As significantly high correlations were detected among the phylogenetic diversity values (*r* > 0.99, *P* < 0.001 for both PD and the standardized PD), one of the complete species trees was randomly selected for downstream analyses.

We detected areas of potential conservation priority (i.e. areas with high phylogenetic diversity but not currently protected) in China and the USA by overlaying phylogenetic diversity hotspots on maps of protected areas. Maps of protected areas in China and the USA were downloaded from the World Database on Protected Areas (WDPA, <https://www.protectedplanet.net/>); the definition of protected areas followed those of the International Union for Conservation of Nature (IUCN) and the Convention on Biological Diversity (CBD). In this study, phylogenetic diversity hotspots are grid cells with the top 5% of PD and the top 5% of standardized PD. To investigate whether hotspots detected in this study encompass areas with high endemism, we analyzed the distribution patterns of phylogenetic endemism (PE) and relative phylogenetic endemism (RPE) in Biodiverse 3.0 [35] following the methods in Mishler *et al*. (2014) [33]. The PE index considered the distribution range of species with each branch divided by the range of all terminal taxa descended from it, while RPE indicated how much observed PE differs from the null expectation [33,36].

Conservation priorities at both the genus and species levels were explored to evaluate the difference in phylogenetic diversity hotspots between the two taxonomic ranks. We also explored the phylogenetic diversity hotspots in China and the USA separately (i.e. top 5% of PD and top 5% of standardized PD within each region) with floras of China and the USA as independent sampling pools while calculating the standardized PD. Such “hotspots” may be of greater importance in making conservation strategies at the country scale.

***Diversity discrepancy in single lineages***

We assessed the generic richness and PD difference between China and the USA for each major lineage, i.e. order [11], to explore which lineages have contributed to the diversity discrepancy between the two regions. Phylogenetic diversity differences were calculated by subtracting the total PD of genera from China and the USA for each order using the “picante” package in R [27]. Spatial patterns of PD for each order were analyzed as described earlier.

***Data availability***

The spatial distribution data, dated phylogeny, and corresponding matrix are available from Dryad (<https://doi.org/10.5061/dryad.12jm63xz3>).

Supplementary Results

***Divergence time estimates and divergence patterns***

We newly generated a phylogeny covering most of the angiosperm genera of China and the USA (93.8% and 88.3% of native genera, respectively). Most grid cells have a high sampling density at the genus level, but the data were limited by a higher proportion of unsampled genera in the western USA (Fig. S2B). Our phylogeny of the angiosperm floras of China and the USA recovered relationships largely congruent with recently published phylogenetic trees of angiosperms at global scale, with only a few exceptions for relationships that remain controversial, such as the position of monocots (Figs 1A and S3) [11,37-39]. Estimations of both familial and generic divergence times are significantly correlated with those obtained from recent global-scale studies, i.e. Li *et al.* (2019) [23], Smith and Brown (2018) [24], and Magallón *et al.* (2015) [22] (Fig. S4C–E and Table S1). Despite some discrepancies between our divergence time estimations and those of previous studies, age deviations for most genera shared between this study and Smith and Brown 2018 [24] are such that 68.1% of genera have an age difference within 10 Myr and 85.2% within 20 Myr (Fig. S4F).

China harbors a higher proportion of genera that originated before the Miocene than the USA (29.9% vs. 23.2%, Table 1). Southern China is a center of diversity for both genera of pre-Miocene origin (highest in Nanling Mountains and southern Yunnan) (Fig. S5A) and recent origin (post-Miocene; highest in Hengduan Mountains and areas adjacent to the border of China with Myanmar, Laos, and Vietnam) (Fig. S5B). In the USA, pre-Miocene genera are mainly distributed in the southeastern coastal areas (North American Coastal Plain and Florida Peninsula) and the southwest (southern California and southern Arizona) (Fig. S5A), while post-Miocene genera are mostly distributed in the southwest (southern California, Arizona, southern Nevada, and western New Mexico) (Fig. S5B).

Temporal divergence analyses show that China has more genera that originated in each geological timespan than the USA (Figs 1B and S6B). Dividing both China and the USA into eastern and western parts, using the 500-mm isoline of annual precipitation in each region (see Supplementary Methods for details), we find that eastern China has many more genera that originated during each geological time interval than western China (Figs 1B and S6B). Within the USA, the east has more genera that originated before the middle Miocene, whereas the west has more genera that originated after that period (Figs 1B and S6B). The herbaceous genera have similar divergence patterns as all genera, but the woody genera in the eastern USA exhibit consistently higher divergence during each geological timespan than those of the western USA (Fig. S6B).

***Spatial diversity patterns***

China harbors 1.4 times more angiosperm genera than the USA (2858 vs. 1992 genera native to the two regions) based on our updated inventory of the two floras. Eastern China (the east side of the 500-mm isoline of annual precipitation), especially the Hengduan Mountains and southern Yunnan, has much higher generic richness than western China or the USA, while western China (west of the 500-mm isoline of annual precipitation) has the lowest value of any region (Fig. 1C–D). Spatial patterns of PD are closely related to those of generic richness (*r* = 0.97, *P* < 0.001), with the eastern USA harboring relatively higher PD than the western USA (two-tailed *t* test, *P* < 0.001; Fig. 1E–F). Both eastern China and the eastern USA showed high standardized PD (Fig. 1G–H) and RPD (Fig. S8A), suggesting that these areas have accumulated many genera with long branches. Low standardized PD (Fig. 1G–H) and RPD (Fig. S8A) occurred in northwestern China and the central to western USA, which indicates a concentration of genera with short branches. In particular, the east-west deviations of standardized PD in both China and the USA are generally congruent with the 500-mm isoline of annual precipitation in each region, which was used to separate the two regions into east and west in this study. Furthermore, herbaceous and woody genera showed similar patterns to all genera in observed PD, but exhibited minor differences in standardized PD and RPD (Figs S8B–C and S10), with herbaceous genera showing the highest values in the northeastern USA.

***Diversity discrepancy in single lineages***

Most clades (51 of 58 orders) have a similar level of sampling density (difference ≤10%) in China and the USA (Table S2), suggesting that PD comparisons between the two regions for the majority of clades are not likely distorted by sampling density. Most lineages (46 of 58 orders) have contributed to the diversity anomaly favoring China (Fig. S3 and Table S2). Lamiales, Asparagales, Malpighiales, Gentianales, Sapindales, Ranunculales, Poales, Fabales, Rosales, and Santalales are the top 10 orders with significantly higher PD in China than the USA. Four orders (i.e. Boraginales, Canellales, Ericales, and Picramniales) with similar sampling density in China and the USA (difference ≤10%) have obvious higher PD (PD difference >100 Myr) in the USA (Fig. S3 and Table S2). However, the contrasting result in Asterales with generic richness higher in the USA but PD favoring China might be impacted by sampling bias between the two regions (sampling ratio: 89.0% in China vs. 66.0% in the USA), as well as possible differences in taxonomic concepts between scientists from the two regions. Spatial patterns of PD for each order showed that orders with higher PD in China generally have hotspots in eastern China (e.g. Hengduan Mountains, southern Yunnan, and Nanling Mountains), while those with higher PD in the USA generally have hotspots in the west (Boraginales and Ericales, Fig. S7).

***Phylogenetic diversity hotspots***

Grid cells with the highest phylogenetic diversity were plotted onto maps of protected areas of China and the USA to investigate their implications for conservation planning. Grid cells with the highest 5% PD overall in the two regions are mainly located in several provinces of southwestern and southern China (e.g. Yunnan, Sichuan, Guizhou, Guangxi, Guangdong, Hunan, Hainan, and Taiwan), while areas with the highest 5% standardized PD values overall in the two regions are located in southern China (e.g. Hunan, Hubei, Jiangxi, Guizhou, Guangxi, and Guangdong) and the eastern USA (e.g. Massachusetts, New York, New Jersey, Virginia, North Carolina, South Carolina, Georgia, and Alabama) (Fig. 1I). Southern China and the eastern USA both show high phylogenetic diversity in our study. Notably, phylogenetic diversity hotspots detected in this study also possess higher phylogenetic endemism (Fig. S12). At the species level, phylogenetic diversity hotspots are concentrated in southern China, e.g. Yunnan, Sichuan, Chongqing, Guizhou, Guangxi, Hunan, Hubei, Guangdong, and Hainan for both PD and standardized PD hotspots, Xizang for PD hotspot, and Taiwan and Jiangxi for standardized PD hotspot (Fig. 1J).

Phylogenetic diversity hotspots within China and the USA, with the taxa of each region treated as an independent sampling pool, are shown in Fig. S11. In China, areas with the highest 5% PD values are more centrally located in the southwest (e.g. Yunnan, Guizhou, and Guangxi), and areas with the highest 5% standardized PD values are concentrated in the southeast (e.g. Hunan, Guangdong, and Jiangxi; Fig. S11A). In the USA, areas with the highest 5% PD values are in both the southeast (e.g. South Carolina, Florida, Alabama, and Mississippi) and the southwest (Nevada, California, and Arizona), while areas with the highest 5% standardized PD values are in the east (Fig. S11A). Phylogenetic diversity hotspots at the species level are largely consistent with those at the genus level, but areas with the highest 5% PD are more concentrated in the Hengduan Mountains of China and the southwestern USA (Fig. S11B).

Limitations and caveats

While our phylogeny includes the most complete sampling for angiosperm genera from China and the USA to date, sampling bias does exist for a few lineages (e.g. Asterales) and the western USA possesses a higher proportion of unsampled genera due to limitation in genetic data. In addition, the two floras of this study are delimitated by administrative boundaries instead of natural barriers, which somehow may have limited our conclusions relative to broader patterns in the northern hemisphere. Besides adding more genetic data from the western USA, future studies with increased sampling (both genetic and distribution data) from the regions adjacent to China (especially those near the Himalayan regions) and the USA (in particular the tropical regions) are recommended to better inform the influence of neighboring floras on regional biodiversity patterns. Such enlarged studies may gain better insights into the broader divergence and diversity patterns of the floras of EA and NA.

Divergence time estimation remains a major challenge for all large-scale phylogenetic analyses, which can suffer from factors such as incomplete sampling, violation of models, and limitation in computational capacity. How to best incorporate age uncertainty and accommodate rate heterogeneity using the penalized likelihood method for large data sets should be explored further [24]. Last, but not least, genera used in this study are actually not biologically comparable entities, but they remain a useful taxonomic unit before complete species-level data are available. A species-level comparison of the two floras has been conducted based on complete-species trees by inserting species without sequence data into a backbone tree according to available taxonomic information, but it remains to be tested how using taxonomy to place many species will impact the phylogenetic diversity patterns [40]. Despite the above limitations and challenges, we are confident in the general patterns yielded through comparisons to the western (and eastern) floras of China and the USA and through comparisons among angiosperm lineages at the order level.

**Supplementary Figures**


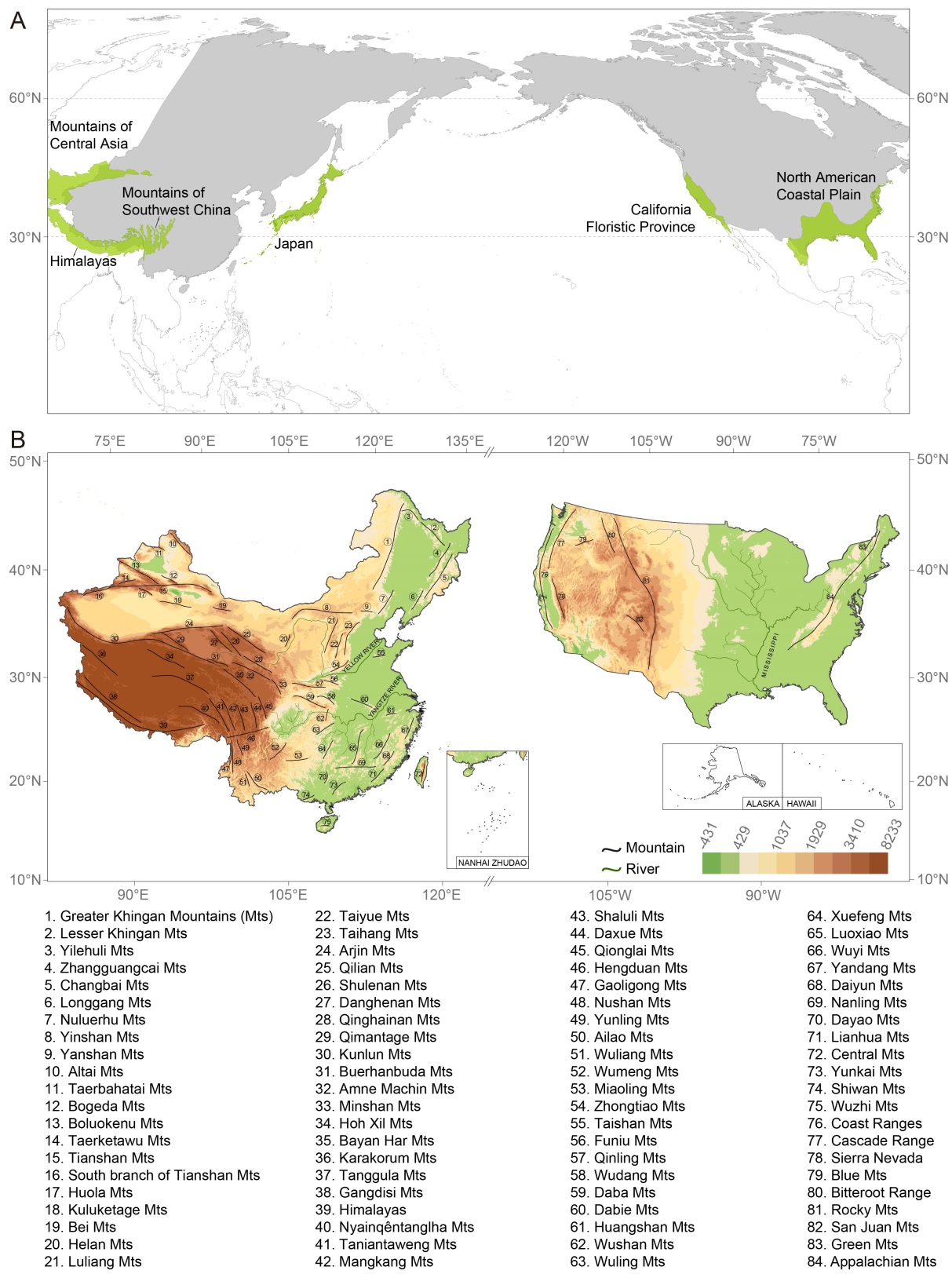


**Figure S1**. Maps showing ranges of (A) EA and NA in this study (gray areas; modified from Qian 2002 [41]) and (B) topography of China and the 48 contiguous states of the USA. Here EA includes the Far East of Russia (roughly east of 80°E), Mongolia, the Korean Peninsula, Japan, and China, and NA includes Canada, Greenland, and the continental USA. Green areas in A show six of the 36 global biodiversity hotspots with four in EA and two in NA [42-44]. Major mountains and rivers in China and the USA are indicated in B. Maps are modified from the National Geomatics Center of China (<http://www.ngcc.cn/>) and Hoffman *et al.* (2016) [45] for biodiversity hotspots. Review drawing number for maps: GS(2021)7893.


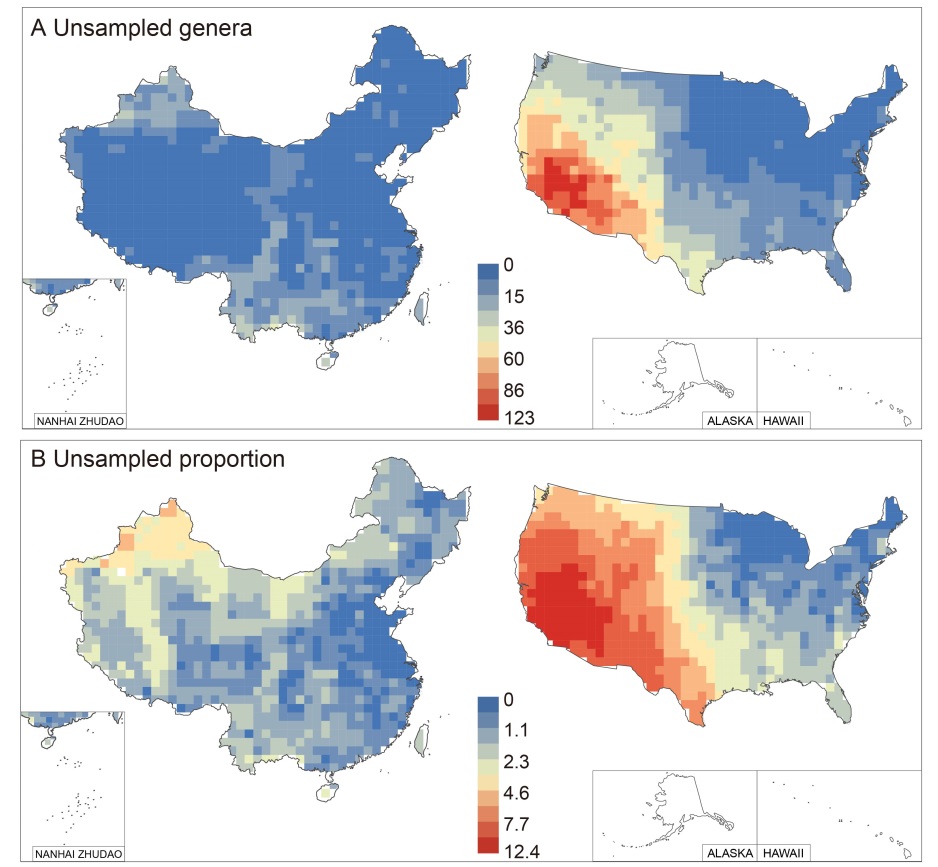


**Figure S2.** Geographic distribution of molecular data gap in China and the USA, showing the distribution patterns of (A) richness and (B) proportion (%) of unsampled genera. The values corresponding to colors represent (A) the number and (B) the proportion of unsampled genera in each grid cell. Review drawing number for maps: GS(2021)7893.


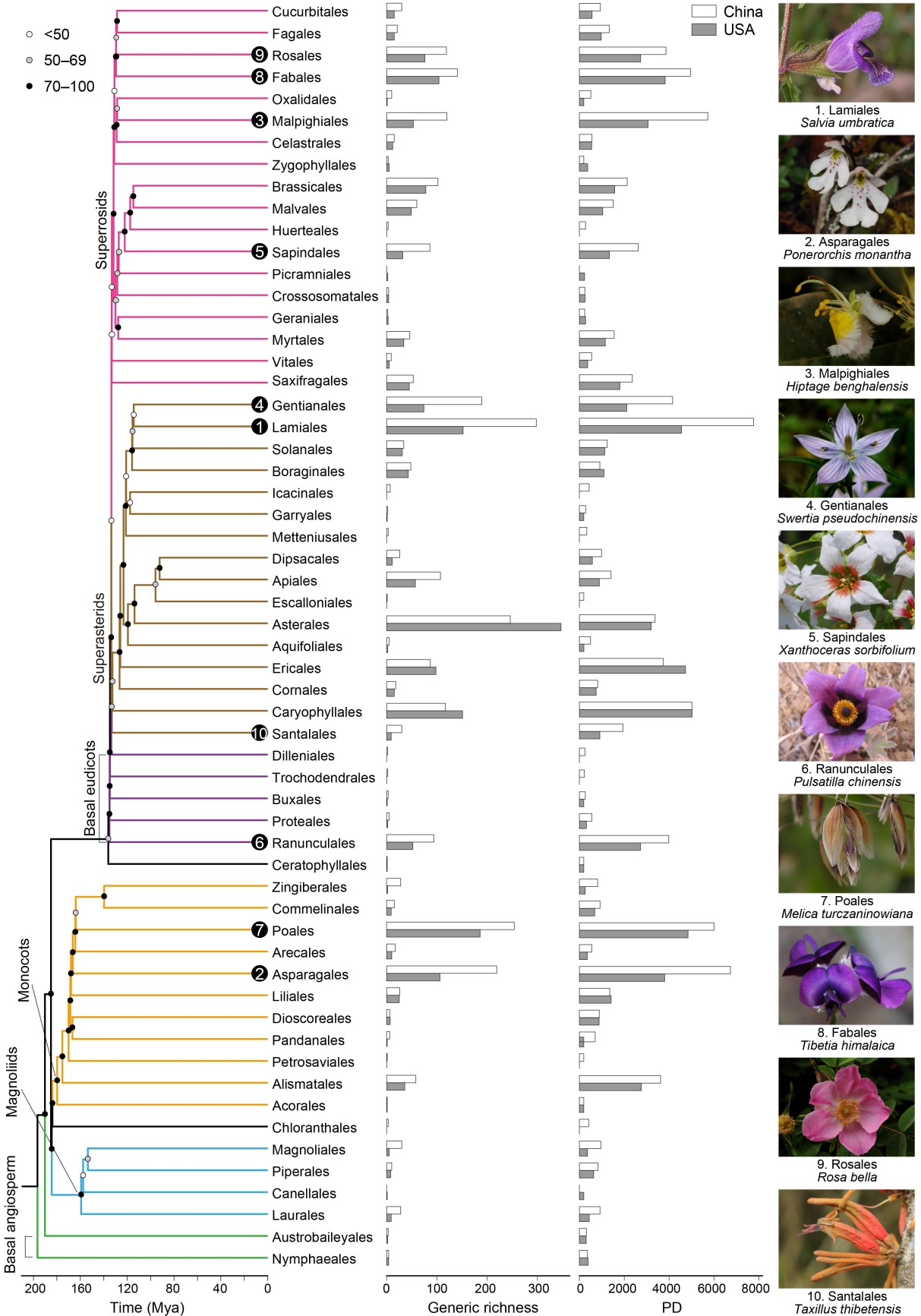


**Figure S3.** A summary order-level chronogram of angiosperms and generic richness and phylogenetic diversity (PD) of each major lineage (order) in China (hollow bar) and the USA (gray bar). The tree was simplified from the dated phylogeny in Fig. 1A. Major clades of angiosperms are shown with different colors. The dot on each node indicates the bootstrap support value from the RAxML analysis. Ten dark circles on the terminals of the tree correspond to the top 10 orders with the highest PD differences between China and the USA; photographs of representative species of these orders are shown on the right (photo credit: Bing Liu).


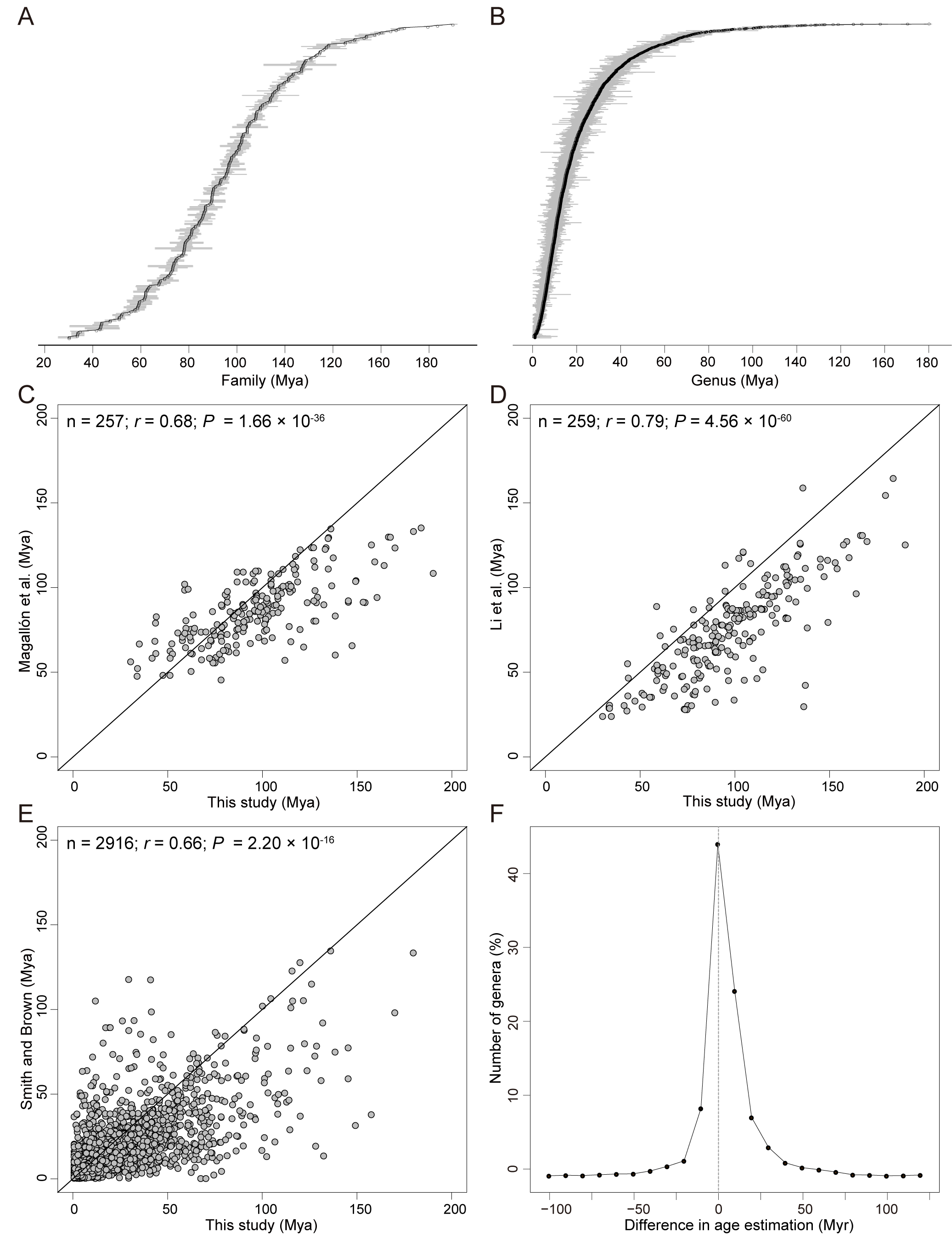


**Figure S4.** Sorted node age estimates using treePL in this study and comparisons of divergence times between our dating and those of recent publications. (A–B) plots of node ages (black circles) and the 95% confidence intervals (gray bars) for (A) families and (B) genera sampled in this study; (C–D) correlation of family ages between this study and (C) Magallón *et al.* (2015) [22] based on three chloroplast genes (*atpB*, *rbcL*, and *matK*) and two nuclear regions (18S and 26S nuclear ribosomal DNA) for 792 angiosperms and (D) Li *et al.* (2019) [23] based on the exons of a chloroplast genome data set of 2351 angiosperms; (E) correlation of genus ages between this study and Smith and Brown (2018) [24] based on a supertree of 79 881 seed plant species; (F) deviation in age estimation for 2916 genera shared between this study and Smith and Brown (2018) [24], showing the proportion of genera with an age difference in each 10-million-year timespan. The solid line is *y* = *x* (C–E).


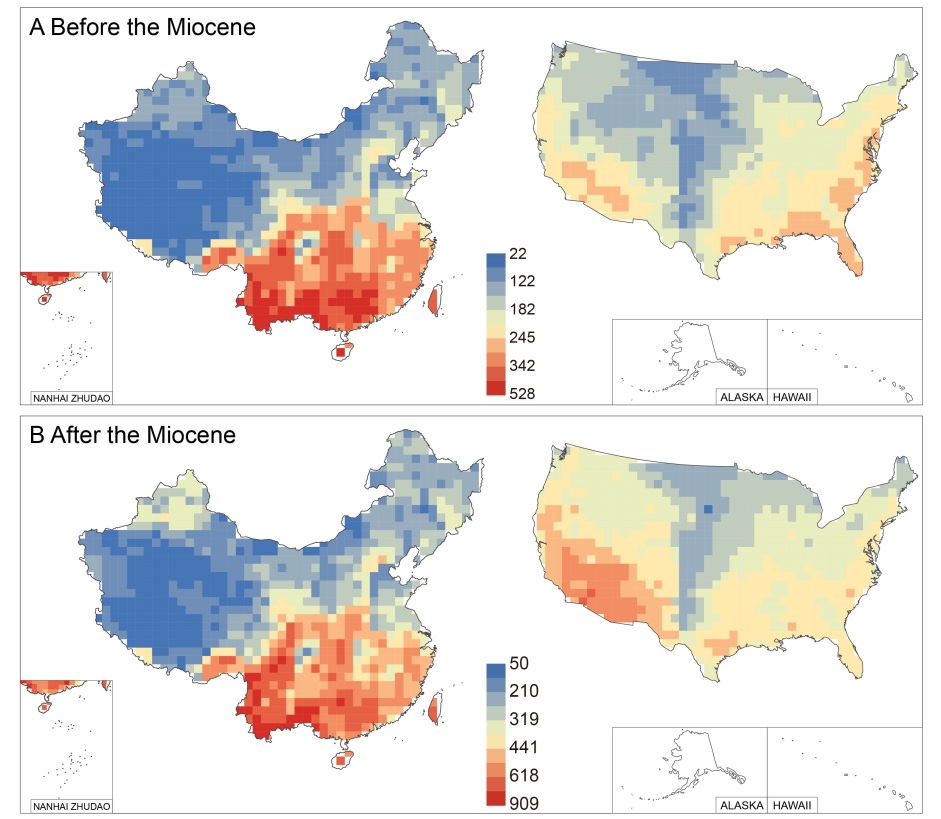


**Figure S5**. Geographic patterns of richness of genera that originated (A) before the Miocene and (B) after the Miocene in China and the USA. The values corresponding to colors represent the number of genera in each grid cell. Review drawing number for maps: GS(2021)7893.


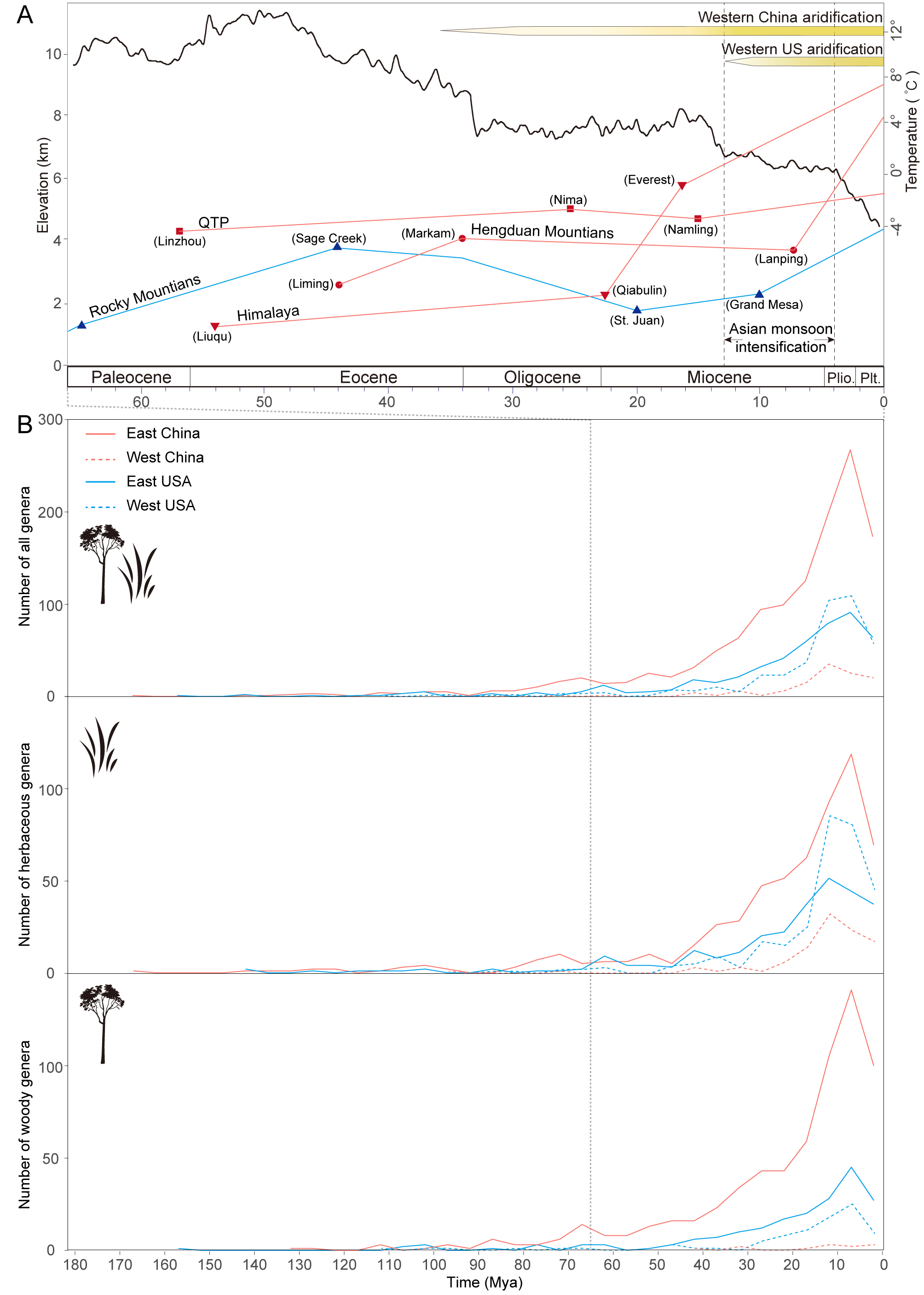


**Figure S6.** Temporal divergence patterns for angiosperm genera in China and the USA. (A) Climatic sequence of events since the Cenozoic, including the estimated deep ocean temperature (black line) modified from Westerhold *et al.* (2020) [46], the aridification in central Asia [47-49] and the western USA [50] (yellow polygons), the intensification of Asian monsoon [51], and the geological sequence of events related to the uplift of the major mountain ranges in China (red lines) and the USA (blue line): the QTP, the Hengduan Mountains, the Himalaya Mountains, and the Rocky Mountains. Selected sites of reconstructed paleoelevation for each mountain are indicated with solid symbols based on the latest evidence (see Table S3); (B) Number of genera that originated during each five-million-year period in eastern China, western China, the eastern USA, and the western USA for all, herbaceous, and woody genera.


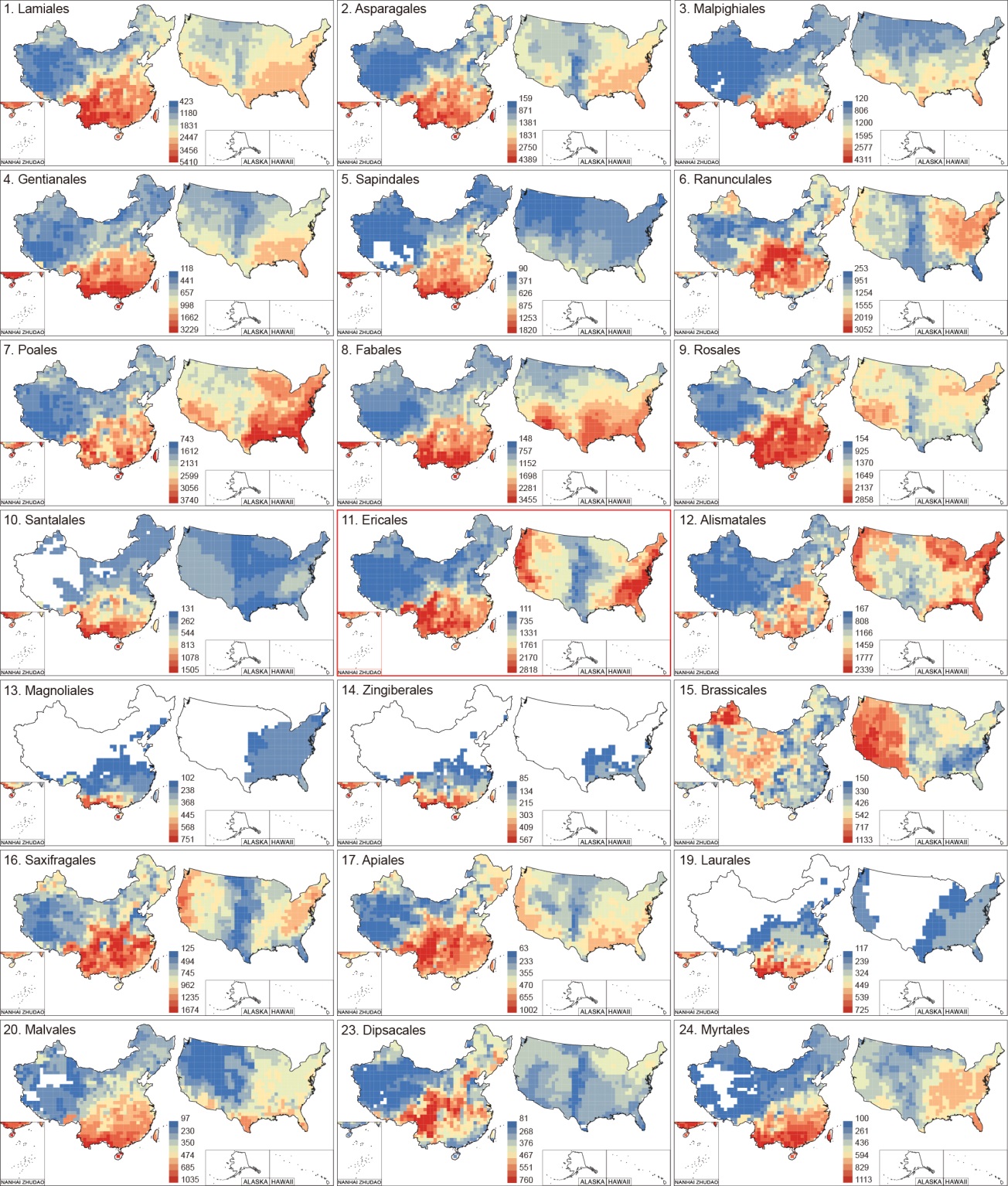


**Figure S7.** Geographic patterns of phylogenetic diversity (PD) for each order in China and the USA. The orders are ranked according to the absolute PD difference between China and the USA in Table S2; the 25 orders with fewer than 10 genera are not shown. Ericales and Boraginales with obvious higher PD in the USA than in China (PD difference >100 million years) are indicated with red frames. The values on the colored bar legends represent PD values in each grid cell. Review drawing number for maps: GS(2021)7893.


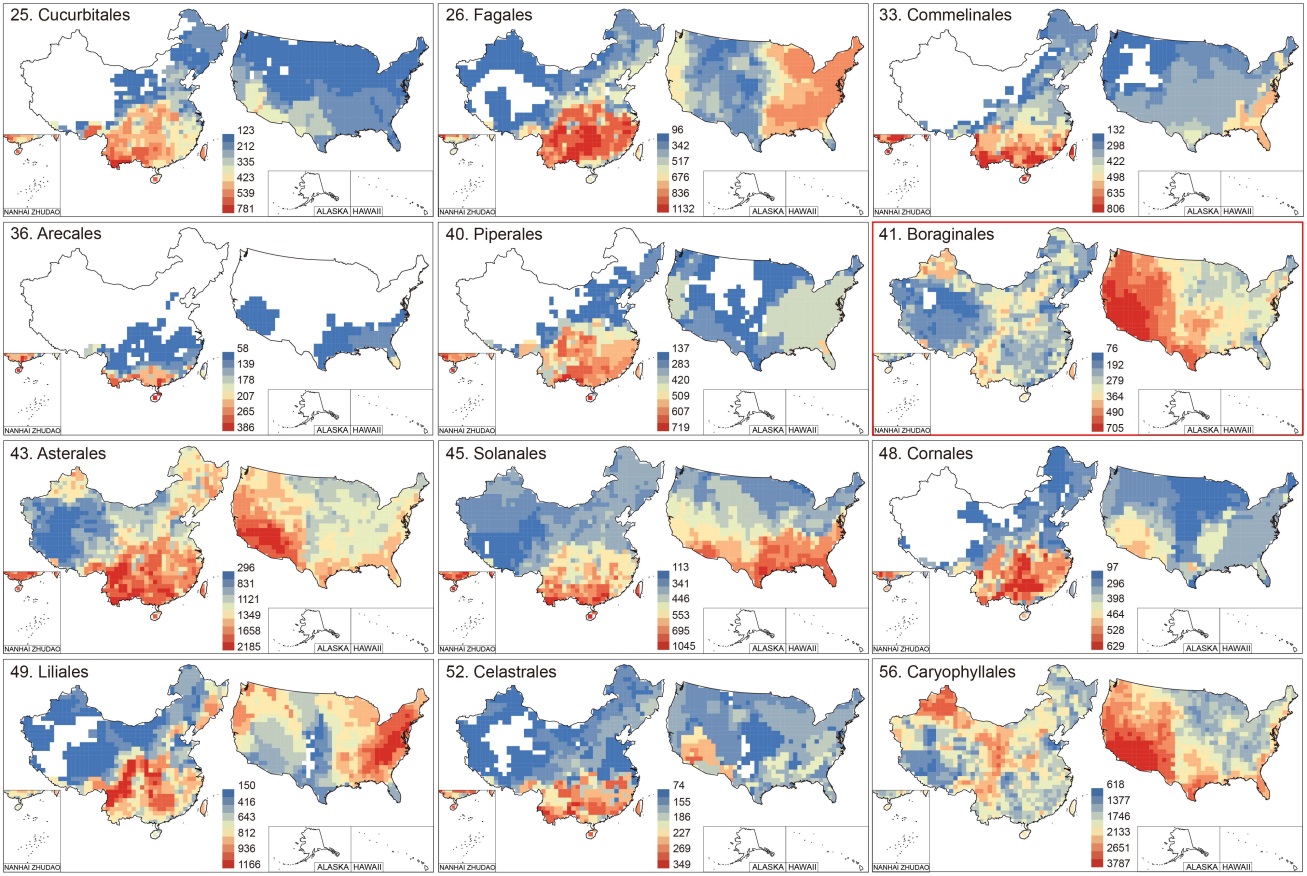


**Figure S7.** Continued.


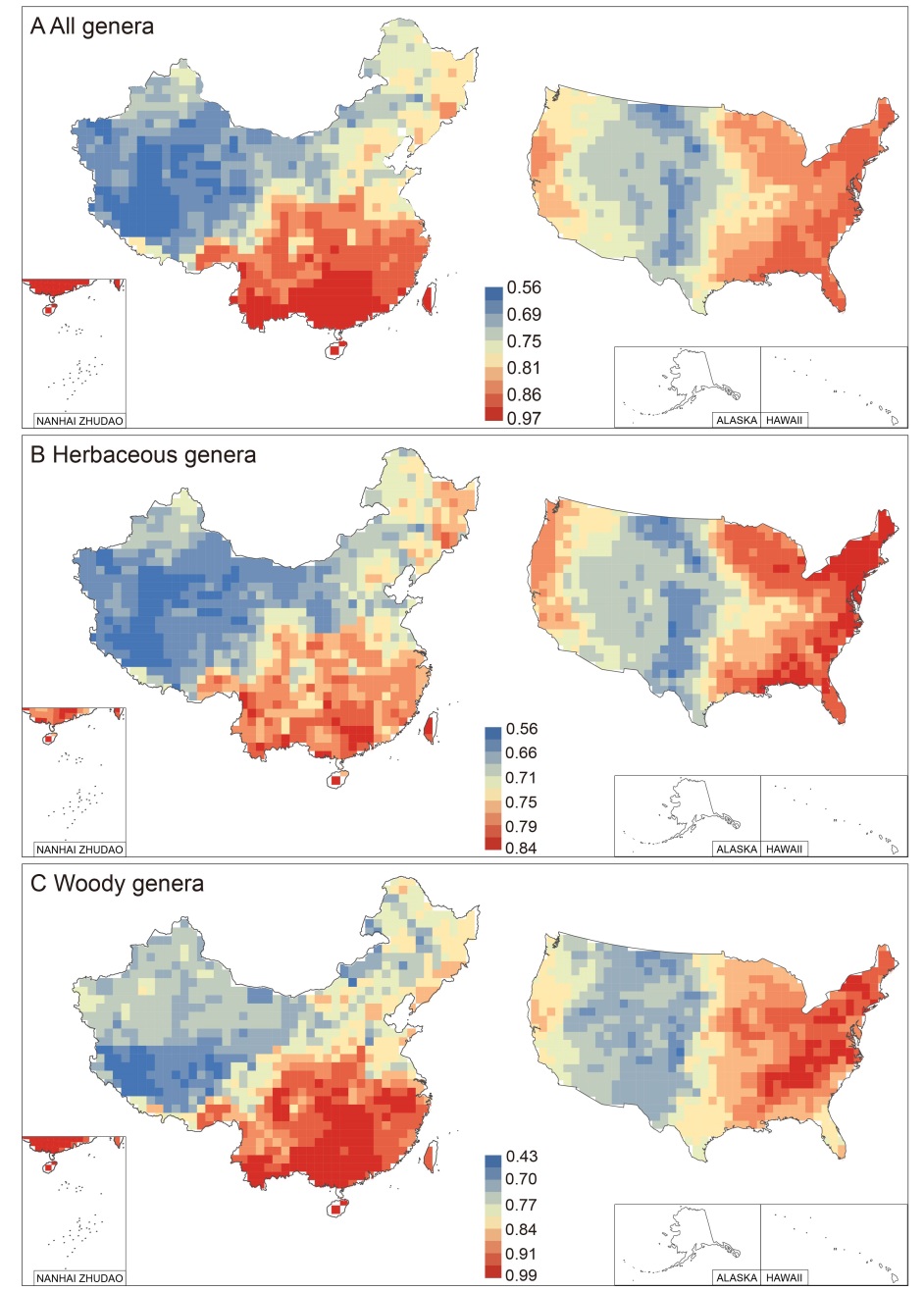


**Figure S8.** Geographic patterns of relative phylogenetic diversity (RPD) for (A) all, (B) herbaceous, and (C) woody genera in China and the USA. The values on the colored bar legends represent RPD values in each grid cell. Review drawing number for maps: GS(2021)7893.


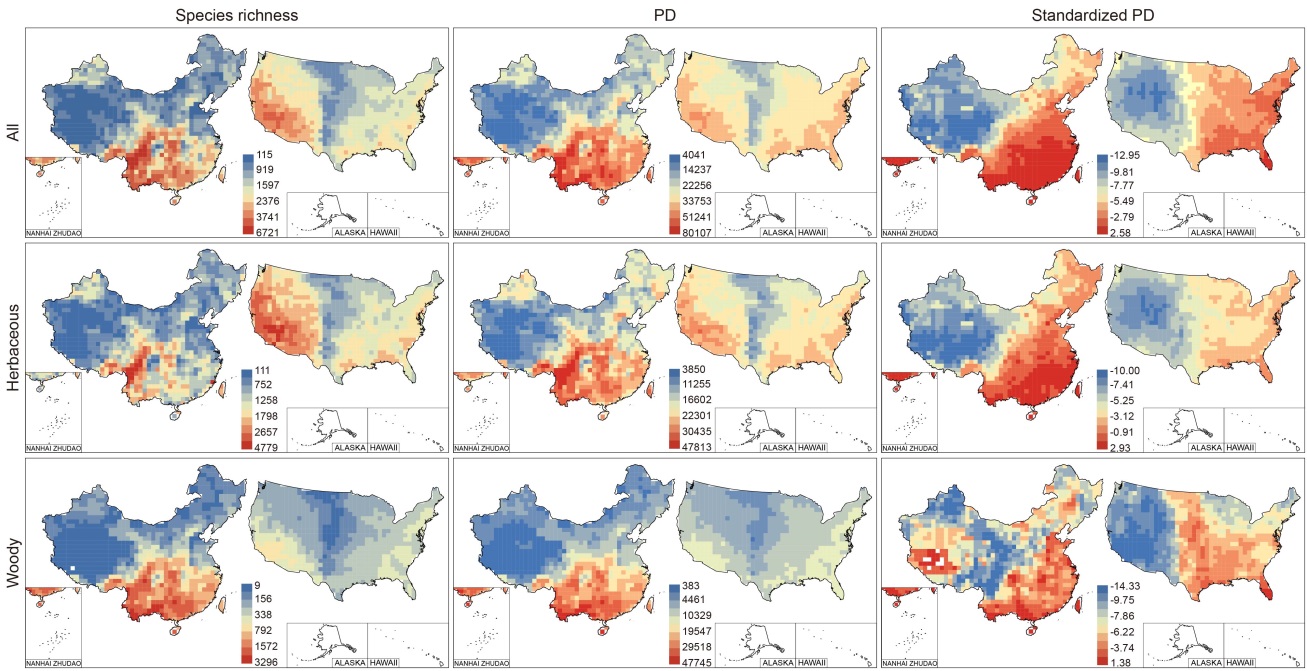


**Figure S9.** Geographic patterns of species richness, phylogenetic diversity (PD), and standardized PD for angiosperms from China and the USA based on a complete species tree generated by “V.PhyloMaker” [34]. The analyses were conducted for all, herbaceous, and woody species separately. The values on the colored bar legends represent generic richness, PD, and standardized PD values in each grid cell. Review drawing number for maps: GS(2021)7893.


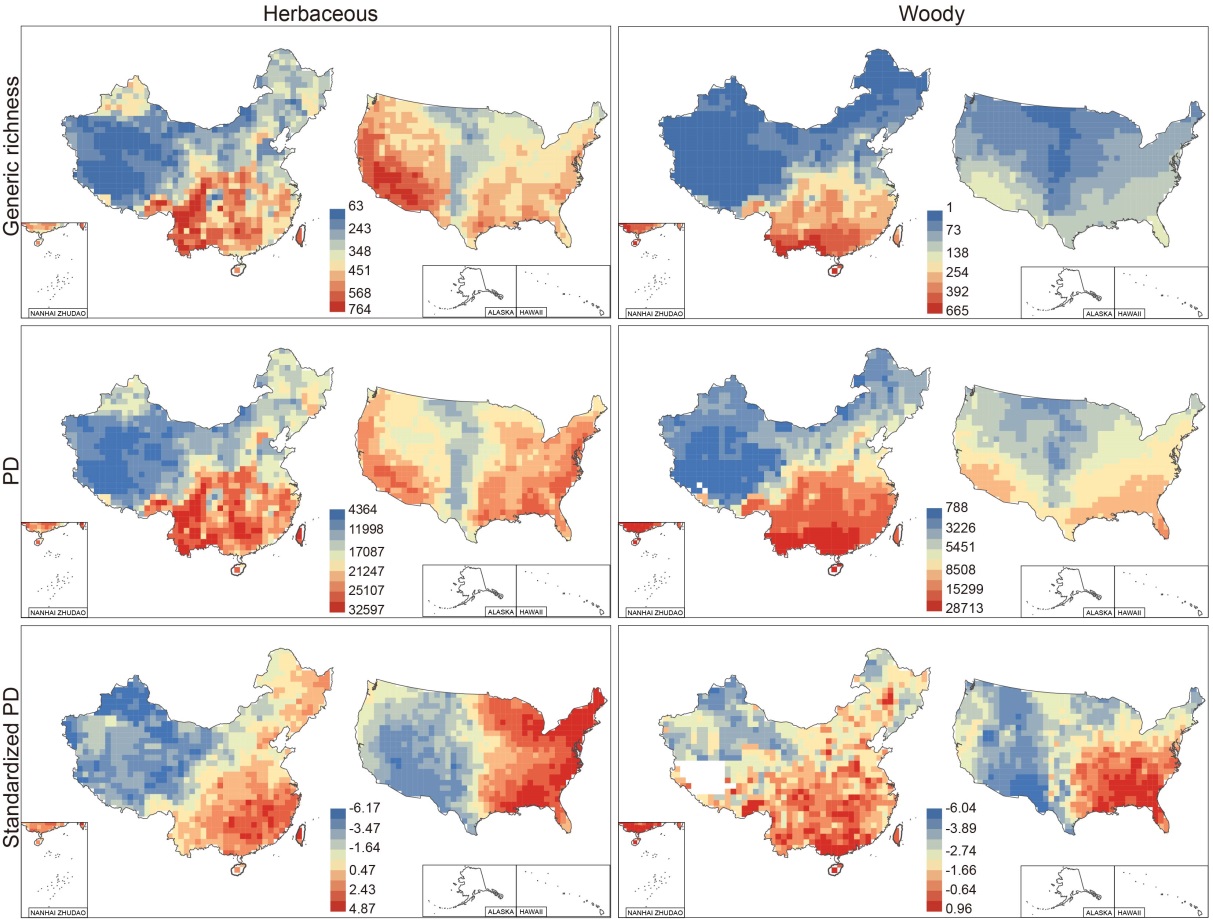


**Figure S10**. Geographic patterns of generic richness, phylogenetic diversity (PD), and standardized PD for herbaceous and woody genera in China and the USA. The values on the colored bar legends represent generic richness, PD, and standardized PD values in each grid cell. Review drawing number for maps: GS(2021)7893.


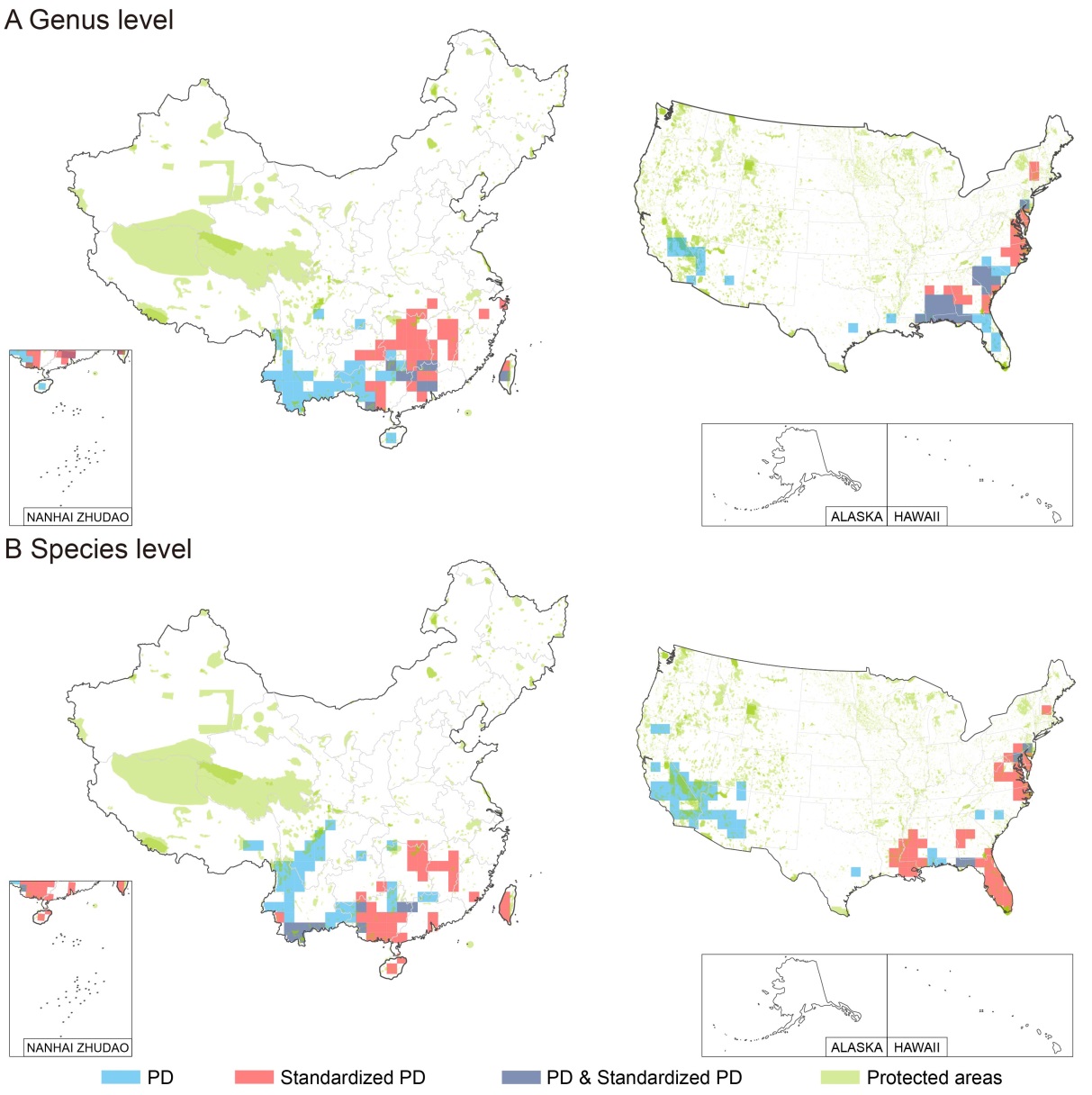


**Figure S11.** Grid cells of top 5% phylogenetic diversity (PD, blue grid cell) and top 5% standardized PD (red grid cell) within China and the USA at the (A) genus and (B) species levels, with protected land areas highlighted in green. The top 5% PD and the top 5% standardized PD are defined as the top 5% of grid cells with the highest PD and standardized PD values within each region. Grid cells with both the top 5% PD and the top 5% standardized PD are indicated in gray. Maps of nature reserves are adapted from the World Database on Protected Areas (WDPA) (<https://www.protectedplanet.net/>). Review drawing number for maps: GS(2021)7893.


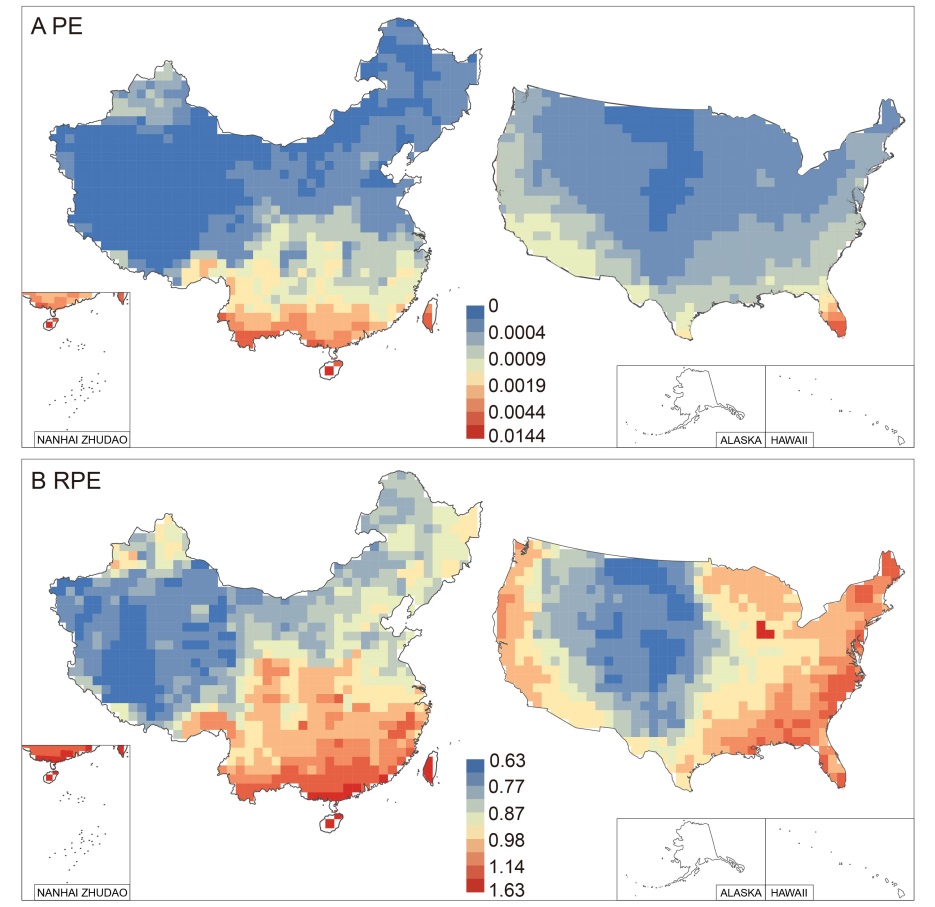


**Figure S12.** Geographic patterns of (A) phylogenetic endemism (PE) and (B) relative phylogenetic endemism (RPE) for angiosperm genera from China and the USA. The values on the colored bar legends represent PE and RPE values in each grid cell. Review drawing number for maps: GS(2021)7893.

**Supplementary Tables**

**Table S1.** Calibrations used for divergence time estimates of the angiosperm floras of China and the USA (MRCA: most recent common ancestor, Mya: million years ago, CG: crown group, SG: stem group).

| Code | Clade | MRCA | min/max | Age | References |
| --- | --- | --- | --- | --- | --- |
|  |  |  |  | (Mya) |  |
| AF | CG Angiosperms | *Nymphaea_alba, Amborella_trichopoda* | min | 136 | Hughes & McDougall, 1987 [52]; Hughes *et al.*, 1991 [53]; Brenner, 1996 [54]; Magallón *et al.*, 2013 [55] |
|  |  |  | max | 209 | Li *et al.*, 2019 [23] |
| AH | SG Nymphaeaceae | *Nymphaea_alba, Cabomba_caroliniana* | min | 108 | Friis *et al.*, 2001 [56], 2009 [57]; Magallón *et al.*, 2015 [22] |
| AI | SG Lauraceae | *Cassytha_filiformis, Illigera_celebica* | min | 104 | Crane *et al.*, 1994 [58]; von Balthazar *et al.*, 2007 [59]; Magallón *et al.*, 2015 [22] |
| AJ | CG eudicots | *Chelidonium_majus, Acalypha_californica* | min | 125 | Leng & Friis, 2003 [60], 2006 [61]; Dilcher *et al.*, 2007 [62]; Magallón *et al.*, 2013 [55] |
| AK | SG eudicots | *Ceratophyllum_demersum, Acalypha_californica* | max | 135.6 | Doyle *et al.*, 1977 [63]; Hughes & McDougall, 1990 [64]; Magallón *et al.*, 2013 [55] |
| AL | SG Buxaceae | *Buxus_microphylla, Tetracera_portobellensis* | min | 100 | Drinnan *et al.*, 1991 [65]; Doyle & Endress, 2010 [66]; Magallón *et al.*, 2013 [55] |
| AM | CG Ericales | *Enkianthus_perulatus, Impatiens_uniflora* | min | 89 | Nixon & Crepet 1993 [67]; Magallón *et al.*, 2013 [55] |
| AN | CG Fagales | *Fagus_engleriana, Carya_cathayensis* | min | 96 | Pacltová, 1966 [68], 1981 [69]; Magallón *et al.*, 2013 [55] |
| AO | CG Rosaceae | *Rosa_transmorrisonensis, Filipendula_vestita* | min | 89.8 | Mai, 1995 [70]; Crepet and Nixon, 1996 [71]; Zhang *et al.*, 2017 [72]; Sun *et al.*, 2019 [73] |
| AQ | SG *Camptotheca* | *Camptotheca_acuminata, Nyssa_sinensis* | min | 33.9 | Manchester & Hickey, 2007 [74]; Xiang *et al.*, 2011 [75] |
| AR | SG *Davidia* | *Davidia_involucrata, Nyssa_sinensis* | min | 56 | Manchester, 2002 [76]; Xiang *et al.*, 2011 [75] |
| AS | SG *Alangium* | *Alangium_chinense, Cornus_sericea* | min | 56 | Farabee & Canright, 1986 [77]; Song *et al.*, 2004 [78]; Xiang *et al.*, 2011 [75] |
| AT | SG *Diplopanax* | *Diplopanax_stachyanthus, Mastixia_euonymoides* | min | 41.2 | Stockey *et al.*, 1998 [79]; Manchester *et al.*, 2009 [80]; Xiang *et al.*, 2011 [75] |
| AU | CG Laurales | *Hernandia_guianensis, Calycanthus_chinensis* | min | 108.8 | Crane *et al.*, 1994 [58]; Bell *et al.*, 2010 [81] |
| AV | CG Hamamelidaceae | *Exbucklandia_populnea, Distylium_myricoides* | min | 84 | Magallón-Puebla *et al.*, 1996 [82]; Magallón *et al.*, 2001 [83]; Bell *et al.*, 2010 [81] |
| AW | CG Poales | *Typha_angustifolia, Pharus_latifolius* | min | 68.1 | Muller, 1981 [84]; Bell *et al.*, 2010 [81] |
| AX | CG Zingiberales | *Orchidantha_chinensis, Zingiber_spectabile* | min | 83.5 | Friis, 1988 [85]; Bell *et al.*, 2010 [81] |
| AY | CG Caryophyllales | *Polygonum_aviculare, Amaranthus_blitoides* | min | 83.5 | Collinson *et al.*, 1993 [86]; Bell *et al.*, 2010 [81] |
| AZ | CG Dilleniaceae | *Dillenia_indica, Tetracera_portobellensis* | min | 51.9 | Collinson *et al.*, 1993 [86]; Bell *et al.*, 2010 [81] |
| BA | CG Vitaceae s.l. | *Vitis_vulpina, Leea_guineensis* | min | 57.9 | Collinson *et al.*, 1993 [86]; Bell *et al.*, 2010 [81] |
| BB | CG Myrtales | *Memecylon_bakerianum, Lythrum_salicaria* | min | 88.2 | Takahashi *et al.*, 1999 [87]; Bell *et al.*, 2010 [81] |
| BC | CG Sapindales | *Nitraria_roborowskii, Biebersteinia_heterostemon* | min | 65 | Knobloch & Mai, 1986 [88]; Bell *et al.*, 2010 [81] |
| BD | CG Fabales | *Cercis_chinensis, Polygala_tenella* | min | 59.9 | Herendeen & Dilcher, 1992 [89]; Bell *et al.*, 2010 [81] |
| BE | SG Iteaceae | *Itea_chinensis, Ribes_maximowiczianum* | min | 89.3 | Hermsen *et al.*, 2003 [90]; Bell *et al.*, 2010 [81] |
|  |  |  | max | 94.6 | Magallón *et al.*, 2015 [22] |
| BF | SG Caprifoliaceae | *Weigela_hortensis, Sambucus_williamsii* | min | 36 | Manchester & Donoghue, 1995 [91]; Bell *et al.*, 2010 [81] |
| BG | SG Bignoniaceae | *Catalpa_ovata, Lantana_involucrata* | min | 49.4 | Wehr & Hopkins, 1994 [92]; Bell *et al.*, 2010 [81] |
| BH | SG *Carya* | *Carya_cathayensis, Pterocarya_tonkinensis* | min | 64 | Manchester & Dilcher, 1997 [93]; Zhang *et al.*, 2013 [94] |
| BI | SG *Carpinus* | *Carpinus_polyneura, Ostrya_virginiana* | min | 59.8 | Manchester *et al.*, 2004 [95]; Sauquet *et al.*, 2012 [96] |
| BJ | SG C*astanopsis* | *Castanopsis_tibetana, Castanea_seguinii* | min | 37.2 | Nixon & Crepet, 1989 [97]; Zhou *et al.*, 2020 [98] |
| BK | CG Stipeae | *Piptatherum_munroi, Pappostipa_speciosa* | min | 34 | MacGinitie, 1953 [99]; Iles *et al.*, 2015 [100]; Burke *et al.*, 2016 [101] |
| BL | CG (BOP clade + PACMAD clade of Poaceae) | *Oryza_rufipogon, Cynodon_transvaalensis* | min | 55 | Crepet & Feldman, 1991[102]; Bouchenak-Khelladi *et al.*, 2010 [103] |
| BM | SG *Neyraudia* | *Neyraudia_reynaudiana, Enneapogon_desvauxii* | min | 19 | Strömberg, 2005 [104]; Prasad *et al.*, 2011 [105] |
| BN | SG *Thalassia* | *Thalassia_hemprichii, Enhalus_acoroides* | min | 33.9 | Lumbert *et al.*, 1984 [106]; Ivany *et al.*, 1990 [107]; Benzecry & Brack-Hanes, 2008 [108]; Iles, 2013 [109] |
| BO | SG Cymodoceaceae | *Halodule_pinifolia, Ruppia_maritima* | min | 33.9 | Iles, 2013 [109] |
| BQ | CG Pandanaceae | *Freycinetia_formosana, Pandanus_tectorius* | min | 33.9 | Iles, 2013 [109] |
| BR | CG (*Phalaenopsis* + *Coelogyne*) | *Phalaenopsis_equestris, Coelogyne_fimbriata* | min | 20 | Conran *et al.*, 2009 [110]; Iles, 2013 [109] |
| BS | CG Cyperaceae | *Mapania_meditensis, Gahnia_baniensis* | min | 47 | Iles, 2013 [109] |
| BT | CG (*Yua* + *Vitis*) | *Yua_austro-orientalis, Vitis_vulpina* | min | 56.8 | Chen & Manchester, 2007 [111]; Wen *et al.*, 2013 [112] |
| BV | CG Schisandraceae | *Schisandra_sphenanthera, Illicium_parvifolium* subsp*. oligandrum* | min | 108 | Friis *et al.*, 1997 [113]; Magallón *et al.*, 2015 [22] |
| BW | SG *Hedyosmum* | *Hedyosmum_arborescens, Sarcandra_glabra* | min | 120.7 | Friis *et al.*, 1994 [114], 1999 [115]; Magallón *et al.*, 2015 [22] |
| BX | SG *Sarcandra* | *Sarcandra_glabra, Chloranthus_spicatus* | min | 99.6 | Crane *et al.*, 1989 [116]; Magallón *et al.*, 2015 [22] |
| BY | SG *Saururus* | *Saururus_chinensis, Gymnotheca_involucrata* | min | 37.2 | Smith & Stockey, 2007 [117]; Magallón *et al.*, 2015 [22] |
| BZ | SG Annonaceae | *Anaxagorea_silvatica, Liriodendron_tulipifera* | min | 87.5 | Takahashi *et al.*, 2008 [118]; Magallón *et al.*, 2015 [22] |
| CA | SG Pontederiaceae | *Monochoria_vaginalis, Murdannia_clarkeana* | min | 40.4 | Mai & Walther, 1978 [119], 1985 [120] ; Wilde, 1989 [121]; Magallón *et al.*, 2015 [22] |
| CB | SG Restionaceae | *Dapsilanthus_ramosus, Flagellaria_indica* | min | 65.5 | Song *et al.*, 2004 [78]; Magallón *et al.*, 2015 [22] |
| CC | SG Poaceae | *Pharus_latifolius, Flagellaria_indica* | min | 55.8 | Muller, 1981 [84]; Magallón *et al.*, 2015 [22] |
| CD | SG Lardizabalaceae | *Kingdonia_uniflora, Sargentodoxa_cuneata* | min | 37.2 | Manchester, 1994 [122]; Magallón *et al.*, 2015 [22] |
| CE | CG Berberidaceae | *Berberis_angulosa, Epimedium_koreanum* | min | 33.9 | Schorn, 1966 [123]; Magallón *et al.*, 2015 [22] |
| CF | CG Sabiaceae | *Sabia_swinhoei, Meliosma_squamulata* | min | 65.5 | Knobloch & Mai, 1986 [88]; Magallón *et al.*, 2015 [22] |
| CG | SG Platanaceae | *Platanus_occidentalis, Heliciopsis_lanceolata* | min | 104 | Crane *et al.*, 1993 [124]; Magallón *et al.*, 2015 [22] |
| CH | SG Loranthaceae | *Taxillus_chinensis, Schoepfia_chinensis* | min | 65.5 | Srivastava, 1966 [125]; Magallón *et al.*, 2015 [22] |
| CI | SG Polygonaceae | *Calligonum_junceum, Plumbago_auriculata* | min | 65.5 | Manchester & O’Leary, 2010 [126]; Magallón *et al.*, 2015 [22] |
| CJ | CG Caryophyllaceae | *Gymnocarpos_decandrus, Drymaria_cordata* | min | 33.9 | Jordan & Macphail, 2003 [127]; Magallón *et al.*, 2015 [22] |
| CK | SG Nyssaceae | *Philadelphus_californicus, Nyssa_sinensis* | min | 65.5 | Knobloch & Mai, 1986 [88]; Magallón *et al.*, 2015 [22] |
| CL | SG Styracaceae | *Berneuxia_thibetica, Huodendron_biaristatum* | min | 48.6 | Mai, 1970 [128]; Magallón *et al.*, 2015 [22] |
| CM | CG Actinidiaceae | *Saurauia_napaulensis, Actinidia_arguta* | min | 83.5 | Keller *et al.*, 1996 [129]; Magallón *et al.*, 2015 [22] |
| CN | SG Apocynaceae | *Alstonia_scholaris, Sebaea_microphylla* | min | 37.2 | MacGinitie, 1969 [130]; Manchester, 1999 [131]; Magallón *et al.*, 2015 [22] |
| CO | CG Gentianaceae | *Sebaea_microphylla, Canscora_diffusa* | min | 33.9 | Crepet & Daghlian, 1981 [132]; Magallón *et al.*, 2015 [22] |
| CP | CG Oleaceae | *Myxopyrum_pierrei, Fontanesia_phillyreoides* | min | 5.33 | Barrón, 1992 [133]; Magallón *et al.*, 2015 [22] |
| CQ | CG Plantaginaceae | *Scoparia_dulcis, Ellisiophyllum_pinnatum* | min | 5.33 | Łańcucka-Środoniowa, 1977 [134]; Magallón *et al.*, 2015 [22] |
| CR | CG Bignoniaceae | *Campsis_grandiflora, Oroxylum_indicum* | min | 38.8 | Meyer & Manchester, 1997 [135]; Magallón *et al.*, 2015 [22] |
| CS | CG Acanthaceae | *Nelsonia_canescens, Avicennia_marina* | min | 28.4 | Reid & Chandler, 1926 [136]; Magallón *et al.*, 2015 [22] |
| CT | SG Paulowniaceae | *Paulownia_tomentosa, Cymbaria_mongolica* | min | 11.6 | Butzmann & Fischer, 1997 [137]; Magallón *et al.*, 2015 [22] |
| CU | CG Lamiaceae | *Siphocranion_macranthum, Callicarpa_arborea* | min | 28.4 | Reid & Chandler, 1926 [136]; Magallón *et al.*, 2015 [22] |
| CV | SG Aquifoliaceae | *Ilex_cornuta, Helwingia_japonica* | min | 61.7 | Mai, 1970 [128]; Magallón *et al.*, 2015 [22] |
| CW | CG Campanulaceae | *Lobelia_sessilifolia, Platycodon_grandiflorus* | min | 5.33 | Łańcucka-Środoniowa, 1977 [134], 1979 [138]; Magallón *et al.*, 2015 [22] |
| CX | CG Menyanthaceae | *Menyanthes_trifoliata, Nymphoides_indica* | min | 5.33 | Łańcucka-Środoniowa, 1979 [138]; Magallón *et al.*, 2015 [22] |
| CY | CG Goodeniaceae | *Goodenia_ovata, Scaevola_taccada* | min | 23.03 | Pocknall, 1982 [139]; Magallón *et al.*, 2015 [22] |
| CZ | SG Asteraceae | *Phagnalon_niveum, Goodenia_varia* | min | 47.6 | Barreda *et al.*, 2012 [140]; Magallón *et al.*, 2015 [22] |
| DA | CG Araliaceae | *Hydrocotyle_javanica, Panax_notoginseng* | min | 37.2 | Manchester, 1994 [122]; Magallón *et al.*, 2015 [22] |
| DB | SG *Weigela* | *Diervilla_lonicera, Weigela_hortensis* | min | 28.4 | Piel, 1971 [141]; Magallón *et al.*, 2015 [22] |
| DC | CG Linnaeoideae | *Linnaea_borealis, Dipelta_yunnanensis* | min | 33.9 | Manchester & Donoghue, 1995 [91]; Magallón *et al.*, 2015 [22] |
| DD | SG Altingiaceae | *Altingia_excelsa, Rhodoleia_championii* | min | 89.3 | Zhou *et al.*, 2001 [142]; Magallón *et al.*, 2015 [22] |
| DE | CG Lythraceae | *Woodfordia_fruticosa, Rotala_ramosior* | min | 70.6 | Estrada-Ruíz *et al.*, 2009 [143]; Magallón *et al.*, 2015 [22] |
| DF | SG Myrtaceae | *Baeckea_frutescens, Crypteronia_paniculata* | min | 83.5 | Eklund, 2003 [144]; Magallón *et al.*, 2015 [22] |
| DG | CG Myrtaceae | *Baeckea_frutescens, Syzygium_buxifolium* | min | 55.8 | Crane *et al.*, 1990 [145]; Pigg *et al.*, 1993 [146]; Magallón *et al.*, 2015 [22] |
| DH | CG Burseraceae | *Canarium_album, Protium_trifoliolatum* | min | 48.6 | Reid & Chandler, 1933 [147]; Collinson, 1983 [148]; Magallón *et al.*, 2015 [22] |
| DI | CG Sapindaceae | *Dimocarpus_longan, Xanthoceras_sorbifolium* | min | 55.8 | Manchester, 2001 [149]; Magallón *et al.*, 2015 [22] |
| DJ | CG Meliaceae | *Chisocheton_cumingianus, Chukrasia_tabularis* | min | 48.6 | Reid & Chandler, 1933 [147]; Magallón *et al.*, 2015 [22] |
| DK | CG Rutaceae | *Harrisonia_perforata, Dictamnus_albus* | min | 65.5 | Knobloch & Mai, 1986 [88]; Magallón *et al.*, 2015 [22] |
| DL | SG Malvaceae | *Corchorus_capsularis, Dipterocarpus_alatus* | min | 55.8 | Carvalho *et al.*, 2011 [150]; Magallón *et al.*, 2015 [22] |
| DM | SG Brassicales | *Bretschneidera_sinensis, Helianthemum_scopulicola* | min | 89.3 | Gandolfo *et al.*, 1998 [151]; Magallón *et al.*, 2015 [22] |
| DN | CG Brassicaceae | *Smelowskia_annua, Berteroella_maximowiczii* | min | 23.03 | Becker, 1961 [152]; Beilstein *et al.*, 2010 [153]; Manchester & O´Leary, 2010 [126]; Magallón *et al.*, 2015 [22] |
| DO | CG Polygalaceae | *Salomonia_cantoniensis, Xanthophyllum_hainanense* | min | 55.8 | Pigg *et al.*, 2008 [154]; Magallón *et al.*, 2015 [22] |
| DP | SG Mimosoideae | *Erythrophleum_fordii, Desmanthus_cooleyi* | min | 48.6 | Crepet & Taylor, 1986 [155]; Magallón *et al.*, 2015 [22] |
| DQ | SG Papilionoideae | *Cladrastis_delavayi, Gymnocladus_chinensis* | min | 55.8 | Herendeen & Wing, 2001 [156]; Magallón *et al.*, 2015 [22] |
| DR | SG Rhamnaceae | *Alphitonia_petriei, Elaeagnus_bockii* | min | 70.6 | Calvillo-Canadell & Cevallos-Ferriz, 2007 [157]; Magallón *et al.*, 2015 [22] |
| DS | CG Rhamnaceae | *Alphitonia_petriei, Ziziphus_obtusifolia* | min | 48.6 | Manchester, 1999 [131]; Magallón *et al.*, 2015 [22] |
| DT | CG Cannabaceae | *Aphananthe_aspera, Gironniera_subaequalis* | min | 65.5 | Knobloch & Mai, 1986 [88]; Magallón *et al.*, 2015 [22] |
| DU | SG *Rhizophora* | *Rhizophora_apiculata, Bruguiera_gymnorhiza* | min | 33.9 | Germeraad, *et al.*, 1968 [158]; Magallón *et al.*, 2015 [22] |
| DV | SG *Populus* | *Salix_lucida, Populus_euphratica* | min | 37.2 | Manchester *et al.*, 1986 [131]; Magallón *et al.*, 2015 [22] |
| DW | SG (*Salix* + *Populus*) | *Idesia_polycarpa, Populus_euphratica* | min | 37.2 | Boucher *et al.*, 2003 [159]; Magallón *et al.*, 2015 [22] |
| DX | SG Clusiaceae | *Garcinia_mangostana, Hypericum_perforatum* | min | 89.3 | Crepet & Nixon, 1998 [160]; Magallón *et al.*, 2015 [22] |
| DY | CG Elaeocarpaceae | *Sloanea_guianensis, Elaeocarpus_reticulatus* | min | 61.7 | Manchester & Kvaček, 2009 [161]; Magallón *et al.*, 2015 [22] |
| DZ | SG *Tripterygium* | *Tripterygium_wilfordii, Celastrus_orbiculatus* | min | 2.58 | Ozaki, 1991 [162]; Magallón *et al.*, 2015 [22] |
| EA | CG Celastraceae | *Mortonia_greggii, Euonymus_alatus* | min | 37.2 | MacGinitie, 1969 [130]; Wolfe, 1977 [163]; Magallón *et al.*, 2015 [22] |
| EB | SG Cucurbitaceae | *Mukia_maderaspatana, Coriaria_nepalensis* | min | 48.6 | Chandler, 1961 [164]; Magallón *et al.*, 2015 [22] |
| ED | CG Juglandaceae | *Pterocarya_hupehensis, Rhoiptelea_chiliantha* | min | 64.4 | Manchester & Dilcher, 1982 [165]; Magallón *et al.*, 2015 [22] |
| EE | CG Fagaceae | *Lithocarpus_henryi, Fagus_engleriana* | min | 37.2 | Crepet & Daghlian, 1980 [166]; Magallón *et al.*, 2015 [22] |
| EF | SG monocots | *Acorus_calamus, Chloranthus_japonicus* | min | 113 | Doyle *et al.*, 2008 [167]; Iles *et al.*, 2015 [100]; Magallón *et al.*, 2015 [22] |
| EG | SG *Caldesia* | *Caldesia_grandis, Sagittaria_guayanensis* | min | 20 | Haggard & Tiffney, 1997 [168]; Chen *et al.*, 2012 [169]; Iles *et al.*, 2015 [100] |
| EH | SG Aponogetonaceae | *Aponogeton_lakhonensis, Scheuchzeria_palustris* | min | 81.13 | Grímsson *et al.*, 2014 [170]; Iles *et al.*, 2015 [100] |
| EI | SG Lasioideae | *Lasia_spinosa, Aglaonema_modestum* | min | 48.7 | Cevallos-Ferriz & Stockey, 1988 [171]; Smith & Stockey, 2003 [172]; Nauheimer *et al.*, 2012 [173]; Iles *et al.*, 2015 [100] |
| EJ | SG Lemnoideae | *Pothoidium_lobbianum, Wolffia_neglecta* | min | 66 | Kvaček, 1995 [174]; Stockey *et al.*, 1997 [175]; Bogner, 2009 [176]; Nauheimer *et al.*, 2012 [173]; Iles *et al.*, 2015 [100] |
| EK | CG Hydrocharitaceae | *Hydrocharis_dubia, Thalassia_hemprichii* | min | 55.9 | Sille *et al.*, 2006 [177]; Chen *et al.*, 2012 [178]; Iles *et al.*, 2015 [100] |
| EL | SG (*Enhalus + Halophila +* *Thalassia*) | *Thalassia_hemprichii, Vallisneria_natans* | min | 38 | Benzecry & Brack-Hanes, 2008 [108]; Iles *et al.*, 2013 [109]; Iles *et al.*, 2015 [100] |
| EM | SG Caryoteae | *Chuniophoenix_nana, Arenga_pinnata* | min | 47.8 | Kar, 1985 [179]; Harley, 2006 [180]; Dransfield *et al.*, 2008 [181]; Iles *et al.*, 2015 [100] |
| EO | SG *Dendrobium* | *Dendrobium_nobile, Bulbophyllum_reptans* | min | 23.2 | Conran *et al.*, 2009 [110]; Iles *et al.*, 2013 [109]; Iles *et al.*, 2015 [100] |
| EP | SG Goodyerinae | *Spiranthes_spiralis, Erythrodes_blumei* | min | 15 | Ramírez *et al.*, 2007 [182]; Guo *et al.*, 2012 [183]; Iles *et al.*, 2015 [100] |
| EQ | SG Hemerocallidoideae | *Hemerocallis_littorea, Eremurus_chinensis* | min | 38 | Conran *et al.*, 2003 [184]; Iles *et al.*, 2013 [109]; Iles *et al.*, 2015 [100] |
| ES | SG Oryzeae | *Bambusa_emeiensis, Oryza_sativa* | min | 66 | Prasad *et al.*, 2011 [185]; Christin *et al.*, 2014 [186]; Iles *et al.*, 2015 [100]; Burke, 2018 [187] |
| ET | SG *Leersia* | *Leersia_oryzoides, Oryza_rufipogon* | min | 30.44 | Walther, 1974 [188]; Walther & Kvaček, 2007 [189]; Iles *et al.*, 2015 [100]; Burke, 2018 [187] |
| EU | SG *Typha* | *Typha_angustifolia, Sparganium_eurycarpum* | min | 51.66 | Grande, 1984 [190]; Iles *et al.*, 2015 [100]; Osozawa *et al.*, 2020 [191] |
| EV | SG Zingiberaceae | *Amomum_villosum, Costus_scaber* | min | 72.1 | Friis, 1988 [85]; Magallón and Sanderson, 2001 [192]; Iles *et al.*, 2015 [100] |
| FE | CG Droseraceae | *Aldrovanda_vesiculosa, Drosera_rotundifolia* | min | 23 | Chandler, 1964 [193]; Mai, 1985 [194]; Dorofeev, 1963 [195]; Friis *et al.*, 2011 [56] |
| FF | CG Schisandraceae s.s. | *Schisandra_sphenanthera, Kadsura_japonica* | min | 33.9 | Gregor, 1981 [196]; Mai & Walther, 1985 [120]; Manchester, 1994 [122]; Mai, 1995 [70]; Denk & Oh, 2005 [197]; Fan *et al.*, 2011 [198]; Friis *et al.*, 2011 [56] |
| FG | CG Primulaceae | *Maesa_tenera, Samolus_valerandi* | min | 66 | Friis *et al.*, 2010 [199]; Friis *et al.*, 2011 [56]; Boucher *et al.*, 2016 [200] |
| FH | CG Smilacaceae | *Smilax_tamnoides, Heterosmilax_chinensis* | min | 56 | Collinson *et al.*, 1993 [86]; Friis *et al.*, 2011 [56] |
| FI | SG Dipterocarpaceae | *Shorea_assamica, Lechea_pulchella* | min | 56 | Crawley, 2001 [201]; Friis *et al.*, 2011[56] |
| FJ | SG Cardiopteridaceae | *Gonocaryum_litorale, Gomphandra_javanica* | min | 52.3 | Magallón *et al.*, 2015 [22] |
| FK | SG Solanaceae | *Withania_somnifera, Stictocardia_macalusoi* | min | 33.9 | Muller, 1981 [84]; Friis *et al.*, 2011 [56] |
| FL | SG Lythraceae | *Ammannia_latifolia, Circaea_alpina* | max | 77.5 | Magallón *et al.*, 2015 [22] |
| FM | SG Arecales | *Wallichia_disticha, Pharus_latifolius* | min | 108.9 | Magallón *et al.*, 2015 [22] |
| FN | SG Eucommiaceae | *Eucommia_ulmoides, Aucuba_japonica* | min | 76.4 | Zanne *et al.*, 2014 [202] |
| FO | SG Myristicaceae | *Horsfieldia_amygdalina, Liriodendron_tulipifera* | max | 101.76 | Magallón *et al.*, 2015 [22] |
| FP | SG Lecythidaceae | *Barringtonia_asiatica, Enkianthus_perulatus* | min | 75.5 | Zanne *et al.*, 2014 [202] |
| FQ | SG Nepenthaceae | *Nepenthes_mirabilis, Ancistrocladus_tectorius* | min | 53.46 | Magallón *et al.*, 2015 [22] |
| FR | SG Haloragaceae | *Gonocarpus_benthamii, Penthorum_chinense* | max | 75.62 | Magallón *et al.*, 2015 [22] |
| FS | SG Cannabaceae | *Cannabis_sativa, Ficus_microcarpa* | max | 81.16 | Magallón *et al.*, 2015 [22] |
| FT | SG Ulmaceae | *Ulmus_americana, Ficus_microcarpa* | max | 88.54 | Magallón *et al.*, 2015 [22] |
| FU | SG Combretaceae | *Terminalia_catappa, Circaea_alpina* | max | 95 | Magallón *et al.*, 2015 [22] |
| FV | CG Polemoniaceae | *Polemonium_caeruleum, Phlox_subulata* | min | 37.2 | Lott *et al.*, 1998 [203]; Magallón *et al.*, 2015 [22] |
| FX | SG Icacinaceae | *Nothapodytes_nimmoniana, Aucuba_himalaica* | min | 65.5 | Wheeler *et al.*, 1987 [204]; Knobloch & Mai 1986 [88]; Magallón *et al.*, 2015 [22] |
| FY | SG Zygophyllaceae | *Krameria_lanceolata, Kallstroemia_parviflora* | min | 23.03 | Weyland 1937 [205]; Manchester & O’Leary 2010 [126]; Magallón *et al.*, 2015 [22] |
| FZ | SG (Juglandaceae+Myricaceae) | *Comptonia_peregrina, Ostrya_rehderiana* | min | 83.5 | Sims *et al.*, 1999 [206]; Magallón *et al.*, 2015 [22] |
| GA | SG Euphorbioideae | *Erismanthus_obliquus, Euphorbia_esula* | min | 37.2 | Crepet & Daghlian 1982 [207]; Magallón *et al.*, 2015 [22] |
| GB | SG Triuridaceae | *Sciaphila_tenella, Croomia_pauciflora* | min | 86.3 | Gandolfo *et al.*, 2002 [208]; Hertweck *et al.*, [209]; Iles *et al.*, 2015 [100] |
| GC | SG *Yucca* | *Yucca_schidigera, Agave_americana* | min | 14.5 | Tidwell & Parker 1990 [210]; Iles *et al.*, 2015 [100]; McKain *et al.*, 2016 [211] |
| GD | SG Hydrangeaceae | *Jamesia_americana, Eucnide_bartonioides* | min | 89.3 | Gandolfo *et al.* 1998 [151]; Magallón *et al.*, 2015 [22] |

**Table S2.** Generic richness and phylogenetic diversity (PD) differences between China and the USA for each order of angiosperms (Myr: million years, /: default value).

| Order | Generic richness in China | Generic richness in the USA | Generic richness differences (China – USA) | Sampling ratio in China (%) | Sampling ratio in the USA (%) | PD in China | PD in the USA | PD differences  (Myr; China – USA) |
| --- | --- | --- | --- | --- | --- | --- | --- | --- |
| Acorales | 1 | 1 | 0 | 100 | 100 | 196 | 196 | 0 |
| Alismatales | 58 | 36 | 22 | 100 | 100 | 3615 | 2760 | 855 |
| Apiales | 107 | 57 | 50 | 84.1 | 80.7 | 1413 | 888 | 525 |
| Aquifoliales | 5 | 2 | 3 | 100 | 100 | 499 | 203 | 296 |
| Arecales | 17 | 10 | 7 | 100 | 100 | 552 | 352 | 199 |
| Asparagales | 219 | 106 | 113 | 94.1 | 97.2 | 6732 | 3800 | 2932 |
| Asterales | 246 | 347 | –101 | 89.0 | 66.0 | 3376 | 3200 | 176 |
| Austrobaileyales | 3 | 2 | 1 | 100 | 100 | 315 | 304 | 11 |
| Boraginales | 48 | 43 | 5 | 85.4 | 86.0 | 911 | 1097 | –186 |
| Brassicales | 102 | 78 | 24 | 89.2 | 96.2 | 2131 | 1576 | 555 |
| Buxales | 3 | 1 | 2 | 100 | 100 | 265 | 196 | 69 |
| Canellales | 0 | 1 | –1 | / | 100 | 0 | 196 | –196 |
| Caryophyllales | 117 | 151 | –34 | 94.0 | 86.8 | 5017 | 5020 | –3 |
| Celastrales | 15 | 12 | 3 | 100 | 100 | 562 | 547 | 16 |
| Ceratophyllales | 1 | 1 | 0 | 100 | 100 | 196 | 196 | 0 |
| Chloranthales | 3 | 0 | 3 | 100 | / | 416 | 0 | 416 |
| Commelinales | 15 | 9 | 6 | 86.7 | 100 | 921 | 684 | 237 |
| Cornales | 18 | 15 | 3 | 100 | 93.3 | 817 | 749 | 68 |
| Crossosomatales | 4 | 4 | 0 | 100 | 100 | 243 | 253 | –11 |
| Cucurbitales | 30 | 15 | 15 | 100 | 100 | 933 | 565 | 369 |
| Dilleniales | 2 | 0 | 2 | 100 | / | 248 | 0 | 248 |
| Dioscoreales | 6 | 7 | –1 | 100 | 100 | 887 | 879 | 8 |
| Dipsacales | 26 | 11 | 15 | 100 | 100 | 988 | 577 | 411 |
| Ericales | 87 | 98 | –11 | 94.3 | 98.0 | 3735 | 4730 | –995 |
| Escalloniales | 1 | 0 | 1 | 100 | / | 196 | 0 | 196 |
| Fabales | 141 | 104 | 37 | 97.2 | 96.2 | 4956 | 3821 | 1135 |
| Fagales | 21 | 15 | 6 | 95.2 | 100 | 1331 | 974 | 357 |
| Garryales | 2 | 1 | 1 | 100 | 100 | 280 | 196 | 84 |
| Gentianales | 189 | 74 | 115 | 92.1 | 91.9 | 4152 | 2111 | 2040 |
| Geraniales | 2 | 3 | –1 | 100 | 100 | 237 | 275 | –38 |
| Huerteales | 3 | 0 | 3 | 100 | / | 273 | 0 | 273 |
| Icacinales | 7 | 0 | 7 | 100 | / | 437 | 0 | 437 |
| Lamiales | 298 | 152 | 146 | 88.6 | 89.5 | 7766 | 4550 | 3216 |
| Laurales | 28 | 9 | 19 | 100 | 100 | 919 | 438 | 481 |
| Liliales | 26 | 25 | 1 | 96.2 | 100 | 1354 | 1414 | –60 |
| Magnoliales | 30 | 5 | 25 | 90.0 | 100 | 956 | 363 | 593 |
| Malpighiales | 120 | 53 | 67 | 95.8 | 100 | 5723 | 3059 | 2664 |
| Malvales | 60 | 49 | 11 | 93.3 | 79.6 | 1511 | 1041 | 470 |
| Metteniusales | 3 | 0 | 3 | 100 | / | 333 | 0 | 333 |
| Myrtales | 46 | 34 | 12 | 97.8 | 79.4 | 1550 | 1153 | 396 |
| Nymphaeales | 4 | 4 | 0 | 100 | 100 | 362 | 388 | –27 |
| Oxalidales | 10 | 1 | 9 | 90.0 | 100 | 511 | 196 | 315 |
| Pandanales | 6 | 1 | 5 | 100 | 100 | 705 | 196 | 509 |
| Petrosaviales | 1 | 0 | 1 | 100 | / | 196 | 0 | 196 |
| Picramniales | 0 | 2 | –2 | / | 100 | 0 | 229 | –229 |
| Piperales | 10 | 8 | 2 | 100 | 75.0 | 834 | 638 | 196 |
| Poales | 254 | 186 | 68 | 97.6 | 94.1 | 5999 | 4840 | 1159 |
| Proteales | 5 | 2 | 3 | 100 | 100 | 559 | 315 | 243 |
| Ranunculales | 94 | 52 | 42 | 97.9 | 90.4 | 3982 | 2724 | 1258 |
| Rosales | 119 | 76 | 43 | 95.8 | 97.4 | 3858 | 2728 | 1130 |
| Santalales | 30 | 9 | 21 | 93.3 | 100 | 1941 | 913 | 1027 |
| Sapindales | 86 | 32 | 54 | 96.5 | 96.9 | 2616 | 1337 | 1280 |
| Saxifragales | 53 | 45 | 8 | 92.5 | 93.3 | 2361 | 1807 | 554 |
| Solanales | 34 | 31 | 3 | 100 | 100 | 1241 | 1129 | 112 |
| Trochodendrales | 2 | 0 | 2 | 100 | / | 217 | 0 | 217 |
| Vitales | 9 | 5 | 4 | 100 | 80.0 | 551 | 370 | 181 |
| Zingiberales | 28 | 2 | 26 | 100 | 100 | 815 | 258 | 556 |
| Zygophyllales | 3 | 5 | –2 | 33.3 | 80.0 | 196 | 371 | –175 |

**Table S3.** Sites used to reconstruct paleoelevation for major mountain ranges in China and the USA in Fig. S6A (Mya: million years ago).

| Mountains | Locality | Age (Mya) | Paleoelevation (m) | Methods | References |
| --- | --- | --- | --- | --- | --- |
| QTP | Linzhou | 60–54 | 4100–4900 | Isotope | Ding *et al.* (2014) [212] |
| QTP | Nima | 26–25 | 4500–5000 | Isotope | DeCelles *et al.* (2007) [213] |
| QTP | Namling | ca. 15 | 3791–5584 | Fossil | Spicer *et al.* (2003) [214] |
| Hengduan Mountains | Liming | 56–33 | 2350–2950 | Isotope | Hoke *et al.* (2014) [215] |
| Hengduan Mountains | Markam | 35–33 | 3590–3900 | Fossil | Su *et al.* (2018) [216] |
| Hengduan Mountains | Lanping | 10–5 | 2850–3750 | Isotope | Hoke *et al.* (2014) [215] |
| Himalaya | Liuqu | ca. 56 | 0–1800 | Plant fossil | Ding *et al.* (2017) [217] |
| Himalaya | Qiabulin | 21–19 | 1400–3200 | Plant fossil | Ding *et al.* (2017) [217] |
| Himalaya | Everest | ca. 16 | 5100–5400 | Isotope | Gébelin *et al.* (2013) [218] |
| Rocky Mountains | Uncertain | ca. 65 | 1000 | Digital model | Bird (1988) [219] |
| Rocky Mountains | Sage Creek | 39–48 | 3700 | Isotope | Mix *et al.* (2011) [220] |
| Rocky Mountains | San Juan | 20 | 1710 | Isotope | Sahagian *et al.* (2002) [221] |
| Rocky Mountains | Grand Mesa | 10 | 2270 | Isotope | Sahagian *et al.* (2002) [221] |

**Supplementary References**

1. Qian H and Ricklefs RE. A comparison of the taxonomic richness of vascular plants in China and the United States. *Am Nat* 1999; **154**: 160–81.

2. Lu LM, Mao LF, Yang T *et al.* Evolutionary history of the angiosperm flora of China. *Nature* 2018; **554**: 234–8.

3. Kartesz JT. The Biota of North America Program (BONAP). Taxonomic Data Center. (<http://www.bonap.net/tdc>). Chapel Hill, North Carolina. 2015.

4. Wu ZY, Raven PH and Hong DY. *Flora of China*. Beijing: Science Press; St. Louis: Missouri Botanical Garden, 1994–2013.

5. Flora of North America Editorial Committee. *Flora of North America, North of Mexico*. New York: Oxford University Press, 1993+.

6. Ricklefs RE and Latham RE. Intercontinental correlation of geographical ranges suggests stasis in ecological traits of relict genera of temperate perennial herbs. *Am Nat* 1992; **139**: 1305–21.

7. Smith SA and Donoghue MJ. Rates of molecular evolution are linked to life history in flowering plants. *Science* 2008; **322**: 86–9.

8. Mazel F, Pennell MW, Cadotte MW *et al.* Prioritizing phylogenetic diversity captures functional diversity unreliably. *Nat Commun* 2018; **9**: 2888.

9. Smiley TM, Title PO, Zelditch ML *et al.* Multi-dimensional biodiversity hotspots and the future of taxonomic, ecological and phylogenetic diversity: A case study of North American rodents. *Global Ecol Biogeogr* 2020; **29**: 516–33.

10. Daru BH, van der Bank M, Maurin O *et al.* A novel phylogenetic regionalization of phytogeographical zones of southern Africa reveals their hidden evolutionary affinities. *J Biogeogr* 2016; **43**: 155–66.

11. APG IV. An update of the Angiosperm Phylogeny Group classification for the orders and families of flowering plants: APG IV. *Bot J Linn Soc* 2016; **181**: 1–20.

12. Chen ZD, Yang T, Lin L *et al.* Tree of life for the genera of Chinese vascular plants. *J Syst Evol* 2016; **54**: 277–306.

13. R Core Team. R: A language and environment for statistical computing. Vienna: R Foundation for Statistical Computing; 2014.

14. Winter DJ. Rentrez: An R package for the NCBI eUtils API. *R J* 2017; **9**: 520–6.

15. Soltis DE and Soltis PS. *Amborella* not a "basal angiosperm"? Not so fast. *Am J Bot* 2004; **91**: 997–1001.

16. Wickett NJ, Mirarab S, Nguyen N *et al.* Phylotranscriptomic analysis of the origin and early diversification of land plants. *Proc Natl Acad Sci USA* 2014; **111**: E4859–68.

17. Katoh K and Standley DM. MAFFT multiple sequence alignment software version 7: Improvements in performance and usability. *Mol Biol Evol* 2013; **30**: 772–80.

18. Hall TA. BioEdit: A user-friendly biological sequence alignment editor and analysis program for Windows 95/98/NT. *Nucl Acids Symp Ser* 1999; **41**: 95–8.

19. Stamatakis A. RAxML version 8: A tool for phylogenetic analysis and post-analysis of large phylogenies. *Bioinformatics* 2014; **30**: 1312–3.

20. Izquierdo-Carrasco F, Smith SA and Stamatakis A. Algorithms, data structures, and numerics for likelihood-based phylogenetic inference of huge trees. *BMC Bioinformatics* 2011; **12**: 470.

21. Smith SA and O'Meara BC. treePL: Divergence time estimation using penalized likelihood for large phylogenies. *Bioinformatics* 2012; **28**: 2689–90.

22. Magallón S, Gómez-Acevedo S, Sánchez-Reyes LL *et al.* A metacalibrated time-tree documents the early rise of flowering plant phylogenetic diversity. *New Phytol* 2015; **207**: 437–53.

23. Li HT, Yi TS, Gao LM *et al.* Origin of angiosperms and the puzzle of the Jurassic gap. *Nat Plants* 2019; **5**: 461–70.

24. Smith SA and Brown JW. Constructing a broadly inclusive seed plant phylogeny. *Am J Bot* 2018; **105**: 302–14.

25. Soltis DE, Morris AB, McLachlan JS *et al.* Comparative phylogeography of unglaciated eastern North America. *Mol Ecol* 2006; **15**: 4261–93.

26. Faith DP. Conservation evaluation and phylogenetic diversity. *Biol Conserv* 1992; **61**: 1–10.

27. Kembel SW, Cowan PD, Helmus MR *et al.* Picante: R tools for integrating phylogenies and ecology. *Bioinformatics* 2010; **26**: 1463–4.

28. Rodrigues ASL, Brooks TM and Gaston KJ. *Phylogeny and Conservation*. Cambridge: Cambridge University Press, 2005.

29. Allen JM, Germain-Aubrey CC, Barve N *et al.* Spatial phylogenetics of Florida vascular plants: The effects of calibration and uncertainty on diversity estimates. *iScience* 2018; **11**: 57–70.

30. Lennon JJ, Koleff P, GreenwooD JJD *et al.* The geographical structure of British bird distributions: Diversity, spatial turnover and scale. *J Anim Ecol* 2001; **70**: 966-79.

31. Koleff P, Gaston KJ and Lennon JJ. Measuring beta diversity for presence-absence data. *J Anim Ecol* 2003; **72**: 367–82.

32. Kreft H and Jetz W. A framework for delineating biogeographical regions based on species distributions. *J Biogeogr* 2010; **37**: 2029–53.

33. Mishler BD, Knerr N, González-Orozco CE *et al.* Phylogenetic measures of biodiversity and neo- and paleo-endemism in Australian *Acacia*. *Nat Commun* 2014; **5**: 4473.

34. Jin Y and Qian H. V.PhyloMaker: An R package that can generate very large phylogenies for vascular plants. *Ecography* 2019; **42**: 1353–9.

35. Laffan SW, Lubarsky E and Rosauer DF. Biodiverse, a tool for the spatial analysis of biological and related diversity. *Ecography* 2010; **33**: 643–7.

36. Rosauer D, Laffan SW, Crisp MD *et al.* Phylogenetic endemism: A new approach for identifying geographical concentrations of evolutionary history. *Mol Ecol* 2009; **18**: 4061–72.

37. Endress PK and Doyle JA. Reconstructing the ancestral flower and its initial specializations. *Am J Bot* 2009; **96**: 22–66.

38. Soltis DE, Smith SA, Cellinese N *et al.* Angiosperm phylogeny: 17 genes, 640 taxa. *Am J Bot* 2011; **98**: 704–30.

39. Zeng LP, Zhang N and Ma H. Advances and challenges in resolving the angiosperm phylogeny. *Biodiv Sci* 2014; **22**: 21–39.

40. Qian H and Jin Y. An updated megaphylogeny of plants, a tool for generating plant phylogenies and an analysis of phylogenetic community structure. *J Plant Ecol* 2016; **9**: 233–9.

41. Qian H. A comparison of the taxonomic richness of temperate plants in East Asia and North America. *Am J Bot* 2002; **89**: 1818–25.

42. Myers N, Mittermeier RA, Mittermeier CG *et al.* Biodiversity hotspots for conservation priorities. *Nature* 2000; **403**: 853–8.

43. Mittermeier RA, Turner WR, Larsen FW *et al.* Global biodiversity conservation: The critical role of hotspots. In: Zachos FE and Habel JC (eds.). *Biodiversity Hotspots—Distribution and Protection of Conservation Priority Areas*. Heidelberg: Springer, 2011, 3–22.

44. Noss RF, Platt WJ, Sorrie BA *et al.* How global biodiversity hotspots may go unrecognized: Lessons from the North American Coastal Plain. *Divers Distrib* 2015; **21**: 236–44.

45. Hoffman M, Koenig K, Bunting G *et al.* *Biodiversity Hotspots (version 2016.1)*. [https://zenodo.org/record/3261807#.YRSZMmnY2Uk](https://zenodo.org/record/3261807%23.YRSZMmnY2Uk) (August 2021, date last accessed).

46. Westerhold T, Marwan N, Drury AJ *et al.* An astronomically dated record of Earth’s climate and its predictability over the last 66 million years. *Science* 2020; **369**: 1383.

47. Ramstein G, Fluteau F, Besse J *et al.* Effect of orogeny, plate motion and land–sea distribution on Eurasian climate change over the past 30 million years. *Nature* 1997; **386**: 788–95.

48. Sun JM, Liu WG, Liu ZH *et al.* Effects of the uplift of the Tibetan Plateau and retreat of Neotethys Ocean on the stepwise aridification of mid-latitude Asian interior. *Bull Chin Acad Sci* 2017; **32**: 951–8.

49. Barbolini N, Woutersen A, Dupont-Nivet G *et al.* Cenozoic evolution of the steppe-desert biome in Central Asia. *Sci Adv* 2020; **6**: eabb8227.

50. Raven PH. The evolution of Mediterranean floras. In: Castri Fd and Mooney HA (eds.). *Mediterranean Ecosystems: Origin and Structure*. New York: Springer, 1973, 213–24.

51. Farnsworth A, Lunt DJ, Robinson SA *et al.* Past East Asian monsoon evolution controlled by paleogeography, not CO_2_. *Sci Adv* 2019; **5**: eaax1697.

52. Hughes NF and McDougall AB. Records of angiospermid pollen entry into the English Early Cretaceous succession. *Rev Palaeobot Palyno* 1987; **50**: 255–72.

53. Hughes NF, McDougall AB and Chapman JL. Exceptional new record of Cretaceous Hauterivian angiospermid pollen from Southern England. *J Micropalaeontol* 1991; **10**: 75–82.

54. Brenner GJ. *Flowering Plant Origin, Evolution, and Phylogeny*. New York: Chapman and Hall, 1996.

55. Magallón S, Hilu KW and Quandt D. Land plant evolutionary timeline: Gene effects are secondary to fossil constraints in relaxed clock estimation of age and substitution rates. *Am J Bot* 2013; **100**: 556–73.

56. Friis EM, Crane PR and Pedersen KR. *Early Flowers and Angiosperm Evolution*. Cambridge: Cambridge University Press, 2011.

57. Friis EM, Pedersen KR, von Balthazar M *et al.* *Monetianthus mirus* gen. et sp. nov., a Nymphaealean flower from the Early Cretaceous of Portugal. *Int J Plant Sci* 2009; **170**: 1086–101.

58. Crane PR, Friis EM and Pedersen KR. Palaeobotanical evidence on the early radiation of magnoliid angiosperms. *Plant Syst Evol (Suppl)* 1994; **8**: 51–72.

59. von Balthazar M, Pedersen KR, Crane PR *et al.* *Potomacanthus lobatus* gen. et sp. nov., a new flower of probable Lauraceae from the Early Cretaceous (Early to Middle Albian) of eastern North America. *Am J Bot* 2007; **94**: 2041–53.

60. Leng Q and Friis EM. *Sinocarpus decussatus* gen. et sp. nov., a new angiosperm with basally syncarpous fruits from the Yixian Formation of Northeast China. *Plant Syst Evol* 2003; **241**: 77–88.

61. Leng Q and Friis EM. Angiosperm leaves associated with *Sinocarpus infructescences* from the Yixian Formation (mid-Early Cretaceous) of NE China. *Plant Syst Evol* 2006; **262**: 173–87.

62. Dilcher DL, Sun G, Ji Q *et al.* An early infructescence *Hyrcantha decussata* (comb. nov.) from the Yixian formation in northeastern China. *Proc Natl Acad Sci USA* 2007; **104**: 9370–4.

63. Doyle JA, Biens P, Doerenkamp A *et al.* Angiosperm pollen from the pre-Albian Lower Cretaceous of equatorial Africa. *Bull Cent Rech Explor Prod Elf-Aquitaine* 1977; **1**: 451–73.

64. Hughes NF and McDougall AB. Barremian-Aptian angiospermid pollen records from southern England. *Rev Palaeobot Palyno* 1990; **65**: 145–51.

65. Drinnan AN, Crane PR, Friis EM *et al.* Angiosperm flowers and tricolpate pollen of buxaceous affinity from the Potomac Group (mid-Cretaceous) of eastern North America. *Am J Bot* 1991; **78**: 153–76.

66. Doyle JA and Endress PK. Integrating Early Cretaceous fossils into the phylogeny of living angiosperms: Magnoliidae and eudicots. *J Syst Evol* 2010; **48**: 1–35.

67. Nixon KC and Crepet WL. Late Cretaceous fossil flowers of ericalean affinity. *Am J Bot* 1993; **80**: 616–23.

68. Pacltová B. Pollen grains of angiosperms in the Cenomanian Peruc Formation in Bohemia. *Palaeobotanist* 1966; **15**: 52–4.

69. Pacltova B. The evolution and distribution of normapolles pollen during the cenophytic. *Rev Palaeobot Palyno* 1981; **35**: 175–208.

70. Mai DH. *Tertiäre Vegetationsgeschichte Europas*. New York: Gustav Fischer Verlag, 1995.

71. Crepet WL and Nixon KC. The fossil history of stamens. In: D'Arcy WG and Keating RC (eds.). *The Anther: Form, Function and Phylogeny*. Cambridge: Cambridge University Press, 1996, 25–57.

72. Zhang SD, Jin JJ, Chen SY *et al.* Diversification of Rosaceae since the Late Cretaceous based on plastid phylogenomics. *New Phytol* 2017; **214**: 1355–67.

73. Sun M, Folk RA, Gitzendanner MA *et al.* Exploring the phylogeny of rosids with a five-locus supermatrix from GenBank. *bioRxiv* (10.1101/694950).

74. Manchester SR and Hickey LJ. Reproductive and vegetative organs of *Browniea* gen. n. (Nyssaceae) from the Paleocene of North America. *Int J Plant Sci* 2007; **168**: 229–49.

75. Xiang QY, Thomas DT and Xiang QP. Resolving and dating the phylogeny of Cornales—Effects of taxon sampling, data partitions, and fossil calibrations. *Mol Phylogenet Evol* 2011; **59**: 123–38.

76. Manchester SR. Leaves and fruits of *Davidia* (Cornales) from the Paleocene of North America. *Syst Bot* 2002; **27**: 368–82.

77. Farabee MJ and Canright JE. Stratigraphic palynology of the lower part of the Lance Formation (Maestrichtian) of Wyoming. *Palaeontogr Abt B* 1986; **199**: 1–89.

78. Song ZC, Wang WM and Huang F. Fossil pollen records of extant angiosperms in China. *Bot Rev* 2004; **70**: 425–58.

79. Stockey RA, Lepage BA and Pigg KB. Permineralized fruits of *Diplopanax* (Cornaceae, Mastixioideae) from the middle Eocene princeton chert of British Columbia. *Rev Palaeobot Palyno* 1998; **103**: 223–34.

80. Manchester SR, Chen ZD, Lu AM *et al.* Eastern Asian endemic seed plant genera and their paleogeographic history throughout the Northern Hemisphere. *J Syst Evol* 2009; **47**: 1–42.

81. Bell CD, Soltis DE and Soltis PS. The age and diversification of the angiosperms re-revisited. *Am J Bot* 2010; **97**: 1296–303.

82. Magallón-Puebla S, Herendeen PS and Endress PK. Allonia decandra: Floral remains of the tribe Hamamelideae (Hamamelidaceae) from Campanian strata of southeastern USA. *Plant Syst Evol* 1996; **202**: 177–98.

83. Magallón S, Herendeen PS and Crane PR. *Androdecidua endressii* gen. et sp. nov., from the Late Cretaceous of Georgia (United States): Further floral diversity in Hamamelidoideae (Hamamelidaceae). *Int J Plant Sci* 2001; **162**: 963–83.

84. Muller J. Fossil pollen records of extant angiosperms. *Bot Rev* 1981; **47**: 1–142.

85. Friis EM. *Spirematospermum chandlerae* sp. nov., an extinct species of Zingiberaceae from the North American Cretaceous. *Tert Res* 1988; **9**: 7–12.

86. Collinson ME, Boulter MC and Holmes PL. Magnoliophyta ('Angiospermae'). In: Benton MJ (ed.) *The fossil record 2*. London: Chapman & Hall, 1993, 809–41.

87. Takahashi M, Crane PR and Ando H. *Esgueiria futabensis* sp. nov., a new angiosperm flower from the Upper Cretaceous (Lower Coniacian) of northeastern Honshu, Japan. *Paleontol Res* 1999; **3**: 81–7.

88. Knobloch E and Mai DH. Monographie der Früchte und Samen in der Kreide Mitteleuropa. *Rozpravy ústredního ústavu geologickénho, Praha* 1986; **47**: 1–219.

89. Herendeen PS and Dilcher DL. *Advances in Legume Systematics IV: The Fossil Record*. London: Royal Botanic Gardens, Kew, 1992.

90. Hermsen EJ, Gandolfo MA, Nixon KC *et al.* *Divisestylus* gen. nov. (aff. Iteaceae), a fossil saxifrage from the Late Cretaceous of New Jersey, USA. *Am J Bot* 2003; **90**: 1373–88.

91. Manchester SR and Donoghue MJ. Winged fruits of Linnaeeae (Caprifoliaceae) in the Tertiary of western North America: *Diplodipelta* gen. nov. *Int J Plant Sci* 1995; **156**: 709–22.

92. Wehr WC and Hopkins DQ. The Eocene orchards and gardens of Republic, Washington. *Washington Geol* 1994; **22**: 27–34.

93. Manchester SR and Dilcher DL. Reproductive and vegetative morphology of *Polyptera* (Juglandaceae) from the Paleocene of Wyoming and Montana. *Am J Bot* 1997; **84**: 649–63.

94. Zhang JB, Li RQ, Xiang XG *et al.* Integrated fossil and molecular data reveal the biogeographic diversification of the eastern Asian-eastern North American disjunct hickory genus (*Carya* Nutt.). *PLoS One* 2013; **8**: e70449.

95. Manchester SR, Pigg KB and Crane PR. *Palaeocarpinus dakotensis* sp. n. (Betulaceae: Coryloideae) and associated staminate catkins, pollen, and leaves from the Paleocene of North Dakota. *Int J Plant Sci* 2004; **165**: 1135–48.

96. Sauquet H, Ho SYW, Gandolfo MA *et al.* Testing the impact of calibration on molecular divergence times using a fossil-rich group: The case of *Nothofagus* (Fagales). *Syst Biol* 2012; **61**: 289–313.

97. Nixon KC and Crepet WL. *Trigonobalanus* (Fagaceae): Taxonomic status and phylogenetic relationships. *Am J Bot* 1989; **76**: 828–41.

98. Zhou WB, Soghigian J and Xiang QY. A new paralog removal pipeline resolves conflict between RAD-seq and enrichment. *bioRxiv* (10.1101/2020.10.26.355248).

99. MacGinitie HD. *Fossil Plants of the Florissant Beds, Colorado*. Washington: Carnegie Institution of Washington, 1953.

100. Iles WJD, Smith SY, Gandolfo MA *et al.* Monocot fossils suitable for molecular dating analyses. *Bot J Linn Soc* 2015; **178**: 346–74.

101. Burke SV, Lin CS, Wysocki WP *et al.* Phylogenomics and plastome evolution of tropical forest grasses (*Leptaspis*, *Streptochaeta*: Poaceae). *Front Plant Sci* 2016; **7**: 1993.

102. Crepet WL and Feldman GD. The earliest remains of grasses in the fossil record. *Am J Bot* 1991; **78**: 1010–4.

103. Bouchenak-Khelladi Y, Verboom GA, Savolainen V *et al.* Biogeography of the grasses (Poaceae): A phylogenetic approach to reveal evolutionary history in geographical space and geological time. *Bot J Linn Soc* 2010; **162**: 543–57.

104. Strömberg CA. Decoupled taxonomic radiation and ecological expansion of open-habitat grasses in the Cenozoic of North America. *Proc Natl Acad Sci USA* 2005; **102**: 11980–4.

105. Prasad V, Strömberg CAE, Leaché AD *et al.* Late Cretaceous origin of the rice tribe provides evidence for early diversification in Poaceae. *Nat Commun* 2011; **2**: 480.

106. Lumbert SH, Hartog CD, Phillips RC *et al.* The occurence of fossil seagrasses in the Avon Park Formation (Late Middle Eocene), Levy County, Florida (U.S.A.). *Aquat Bot* 1984; **20**: 121–9.

107. Ivany LC, Portell RW and Jones DS. Animal-plant relationships and paleobiogeography of an Eocene seagrass community from Florida. *Palaios* 1990; **5**: 244–58.

108. Benzecry A and Brack-Hanes SD. A new hydrocharitacean seagrass from the Eocene of Florida. *Bot J Linn Soc* 2008; **157**: 19–30.

109. Iles WJD. The phylogeny and evolution of two ancient lineages of aquatic plants. *Doctoral thesis*. University of British Columbia 2013.

110. Conran JG, Bannister JM and Lee DE. Earliest orchid macrofossils: Early Miocene *Dendrobium* and *Earina* (Orchidaceae: Epidendroideae) from New Zealand. *Am J Bot* 2009; **96**: 466–74.

111. Chen I and Manchester SR. Seed morphology of modern and fossil *Ampelocissus* (Vitaceae) and implications for phytogeography. *Am J Bot* 2007; **94**: 1534–53.

112. Wen J, Xiong ZQ, Nie ZL *et al.* Transcriptome sequences resolve deep relationships of the grape family. *PLoS One* 2013; **8**: e74394.

113. Friis EM, Crane PR and Pedersen KR. *Anacostia*, a new basal angiosperm from the Early Cretaceous of North America and Portugal with trichotomocolpate/monocolpate pollen. *Grana* 1997; **36**: 225–44.

114. Friis EM, Pedersen KR and Crane PR. Angiosperm floral structures from the Early Cretaceous of Portugal. *Plant Syst Evol (Suppl)* 1994; **8**: 31–49.

115. Friis EM, Pedersen KR and Crane PR. Early angiosperm diversification: The diversity of pollen associated with angiosperm reproductive structures in Early Cretaceous floras from Portugal. *Ann Missouri Bot Gard* 1999; **86**: 259–96.

116. Crane PR, Friis EM and Pedersen KR. Reproductive structure and function in Cretaceous Chloranthaceae. *Plant Syst Evol* 1989; **165**: 211–26.

117. Smith SY and Stockey RA. Establishing a fossil record for the perianthless Piperales: *Saururus tuckerae* sp. nov. (Saururaceae) from the Middle Eocene Princeton Chert. *Am J Bot* 2007; **94**: 1642–57.

118. Takahashi M, Friis EM, Uesugi K *et al.* Floral evidence of Annonaceae from the Late Cretaceous of Japan. *Int J Plant Sci* 2008; **169**: 908–17.

119. Mai DH and Walther H. Floren der Haselbacher Serie im Weisselster-Becken (Bezirk Leipzig, DDR). *Abh Sta Mus Mineral Geol Dre* 1978; **28**: 1–200.

120. Mai DH and Walther H. Die obereozänen Floren des Weisselster-Beckens (Berzik Leipzig, DDR). *Abh Sta Mus Mineral Geol Dre* 1985; **33**: 1–220.

121. Wilde V. Untersuchungen zur Systematik der Blattreste aus dem Mitteleozän der Grube Messel bei Darmstadt (Hessen, Bundesrepublik Deutschland). *Cour Forschungsinst Senckenb* 1989; **115**: 1–213.

122. Manchester SR. Fruits and seeds of the middle Eocene Nut Beds flora, Clarno Formation, Oregon. *Palaeontogr Am* 1994; **58**: 1–205.

123. Schorn H. *Revision of the Fossil Species of Mahonia from North America*. Berkeley: University of California Press, 1966.

124. Crane PR, Pedersen KR, Friis EM *et al.* Early Cretaceous (Early to Middle Albian) platanoid inflorescences associated with *Sapindopsis* leaves from the Potomac Group of Eastern North America. *Syst Bot* 1993; **18**: 328–44.

125. Srivastava S. Upper Cretaceous microflora (Maestrichtian) from Scollard, Alberta, Canada. *Pollen Spores* 1966; **8**: 497–552.

126. Manchester SR and O'Leary EL. Phylogenetic distribution and identification of fin-winged fruits. *Bot Rev* 2010; **76**: 1–82.

127. Jordan GJ and Macphail MK. A Middle-Late Eocene inflorescence of Caryophyllaceae from Tasmania, Australia. *Am J Bot* 2003; **90**: 761–8.

128. Mai DH. Subtropische Elemente im Europäischer Tertiär I: Die Gattungen *Gironiera*, *Sarcococa*, *Illicium*, *Evodia*, *Ilex*, *Mastixia*, *Alangium*, *Symplocos* und *Rehderodendron*. *Paläontol Abh Abt B, Paläobot* 1970; **3**: 441–503.

129. Keller JA, Herendeen PS and Crane PR. Fossil flowers and fruits of the Actinidiaceae from the Campanian (Late Cretaceous) of Georgia. *Am J Bot* 1996; **83**: 528–41.

130. MacGinitie HD. The Eocene Green River flora of northwestern Colorado and northeastern Utah. *Univ Calif Publ Geol Sci* 1969; **83**: 133–40.

131. Manchester SR. Biogeographical relationships of North American Tertiary floras. *Ann Missouri Bot Gard* 1999; **86**: 472–522.

132. Crepet WL and Daghlian CP. Lower Eocene and Paleocene Gentianaceae: Floral and palynological evidence. *Science* 1981; **214**: 75–7.

133. Barrón E. Presencia de *Fraxinus excelsior* Linne (Oleaceae, Gentianales) en el Mioceno superior de la depresión Ceretana: Implicaciones tafonóicas y paleoecológicas. *Rev Esp Paleontol* 1992; **7**: 101–8.

134. Łańcucka-Środoniowa M. New herbs described from the Tertiary of Poland. *Acta Palaeobot* 1977; **18**: 37–44.

135. Meyer HW and Manchester SR. *Oligocene Bridge Creek Flora of the John Day Formation, Oregon*. Berkeley: University of California Press, 1997.

136. Reid EM and Chandler MEJ. *Catalogue of Cainozoic Plants in the Department of Geology I: The Brembridge Flora*. London: British Museum of Natural History, 1926.

137. Butzmann R and Fischer TC. Description of the fossil fruit *Paulownia inopinata* nov. spec. from the Middle Miocene of Unterwohlbach (Bavaria) and other possible occurrences of the genus in the Tertiary. *Doc Nat* 1997; **115**: 1–13.

138. Łańcucka-Środoniowa M. Macroscopic plant remains from the freshwater Miocene of the Nowy Sacz basin (West Carpathians, Poland). *Acta Palaeobot* 1979; **20**: 3–117.

139. Pocknall DT. Palynology of late Oligocene Pomahaka Estuarine Bed sediments, Waikoikoi, Southland, New Zealand. *New Zeal J Bot* 1982; **20**: 263–87.

140. Barreda VD, Palazzesi L, Katinas L *et al.* An extinct Eocene taxon of the daisy family (Asteraceae): Evolutionary, ecological and biogeographical implications. *Ann Bot* 2012; **109**: 127–34.

141. Piel KM. Palynology of Oligocene sediments from central British Columbia. *Can J Bot* 1971; **49**: 1885–920.

142. Zhou ZK, Crepet WL and Nixon KC. The earliest fossil evidence of the Hamamelidaceae: Late Cretaceous (Turonian) inflorescences and fruits of Altingioideae. *Am J Bot* 2001; **88**: 753–66.

143. Estrada-Ruiz E, Calvillo-Canadell L and Cevallos-Ferriz SRS. Upper Cretaceous aquatic plants from Northern Mexico. *Aquat Bot* 2009; **90**: 282–8.

144. Eklund H. First Cretaceous flowers from Antarctica. *Rev Palaeobot Palyno* 2003; **127**: 187–217.

145. Crane PR, Manchester SR and Dilcher DL. A preliminary survey of fossil leaves and well-preserved reproductive structures from the Sentinel Butte Formation (Paleocene) near Almont, North Dakota. *Fieldiana Geol* 1990; **20**: 1–63.

146. Pigg KB, Stockey RA and Maxwell SL. *Paleomyrtinaea*, a new genus of permineralized myrtaceous fruits and seeds from the Eocene of British Columbia and Paleocene of North Dakota. *Can J Bot* 1993; **71**: 1–9.

147. Reid EM and Chandler MEJ. *London Clay Flora*. London: British Museum of Natural History, 1933.

148. Collinson ME. *Fossil Plants of the London Clay I: Field Guide to Fossils*. London: Palaeontological Association, 1983.

149. Manchester SR. Leaves and fruits of *Aesculus* (Sapindales) from the Paleocene of North America. *Int J Plant Sci* 2001; **162**: 985–98.

150. Carvalho MR, Herrera FA, Jaramillo CA *et al.* Paleocene Malvaceae from northern South America and their biogeographical implications. *Am J Bot* 2011; **98**: 1337–55.

151. Gandolfo MA, Nixon KC and Crepet WL. *Tylerianthus crossmanensis* gen. et sp. nov. (aff. Hydrangeaceae) from the Upper Cretaceous of New Jersey. *Am J Bot* 1998; **85**: 376–86.

152. Becker HF. *Oligocene plants from the Upper Ruby River Basin, southwestern Montana*. New York: Geological Society of America, 1961.

153. Beilstein MA, Nagalingum NS, Clements MD *et al.* Dated molecular phylogenies indicate a Miocene origin for *Arabidopsis thaliana*. *Proc Natl Acad Sci USA* 2010; **107**: 18724–8.

154. Pigg KB, DeVore ML and Wojciechowski MF. *Paleosecuridaca curtisii* gen. et sp. nov., Securidaca-like samaras (Polygalaceae) from the Late Paleocene of North Dakota and their significance to the divergence of families within the Fabales. *Int J Plant Sci* 2008; **169**: 1304–13.

155. Crepet WL and Taylor DW. Primitive mimosoid flowers from the Paleocene-Eocene and their systematic and evolutionary implications. *Am J Bot* 1986; **73**: 548–63.

156. Herendeen PS and Wing S. *Papilionoid Legume Fruits and Leaves from the Paleocene of Northwestern Wyoming*. St. Louis: Botanical Society of America, 2001.

157. Calvillo-Canadell L and Cevallos-Ferriz SRS. Reproductive structures of Rhamnaceae from the Cerro del Pueblo (Late Cretaceous, Coahuila) and Coatzingo (Oligocene, Puebla) Formations, Mexico. *Am J Bot* 2007; **94**: 1658–69.

158. Germeraad JH, Hopping CA and Muller J. Palynology of Tertiary sediments from tropical areas. *Rev Palaeobot Palyno* 1968; **6**: 189–348.

159. Boucher LD, Manchester SR and Judd WS. An extinct genus of Salicaceae based on twigs with attached flowers, fruits, and foliage from the Eocene Green River Formation of Utah and Colorado, USA. *Am J Bot* 2003; **90**: 1389–99.

160. Crepet WL and Nixon KC. Fossil Clusiaceae from the Late Cretaceous (Turonian) of New Jersey and implications regarding the history of bee pollination. *Am J Bot* 1998; **85**: 1122–33.

161. Manchester SR and Kvaček Z. Fruits of *Sloanea* (Elaeocarpaceae) in the Paleogene of North America and Greenland. *Int J Plant Sci* 2009; **170**: 941–50.

162. Ozaki K. *Late Miocene and Pliocene Floras in Central Honshu, Japan*. Yokohama: Kanagawa Prefectural Museum, 1991.

163. Wolfe JA. Paleogene floras from the Gulf of Alaska region. *US Geol Surv Prof Pap* 1977; **997**: 1–108.

164. Chandler MEJ. *The Lower Tertiary Floras of Southern England I: Paleocene Floras, London Clay Flora (Suppl.)*. London: British Museum of Natural History, 1961.

165. Manchester SR and Dilcher DL. Pterocaryoid fruits (Juglandaceae) in the Paleogene of North America and their evolutionary and biogeographic significance. *Am J Bot* 1982; **69**: 275–86.

166. Crepet WL and Daghlian CP. Castaneoid inflorescences from the Middle Eocene of Tennessee and the diagnostic value of pollen (at the subfamily level) in the Fagaceae. *Am J Bot* 1980; **67**: 739–57.

167. Doyle JA, Endress PK and Upchurch GR. Early Cretaceous monocots: A phylogenetic evaluation. *Acta Mus Natl Pragae, Ser B Hist Nat* 2008; **64**: 59–87.

168. Haggard K and Tiffney B. The flora of the early Miocene Brandon Lignite, Vermont, USA. VIII. *Caldesia* (Alismataceae). *Am J Bot* 1997; **84**: 239–52.

169. Chen LY, Chen JM, Gituru RW *et al.* Generic phylogeny and historical biogeography of Alismataceae, inferred from multiple DNA sequences. *Mol Phylogenet Evol* 2012; **63**: 407–16.

170. Grímsson F, Zetter R, Halbritter H *et al.* *Aponogeton* pollen from the Cretaceous and Paleogene of North America and West Greenland: Implications for the origin and palaeobiogeography of the genus. *Rev Palaeobot Palynol* 2014; **200**: 161–87.

171. Cevallos-Ferriz S and Stockey RA. Permineralized fruits and seeds from the Princeton Chert (Middle Eocene) of British Columbia: Araceae. *Am J Bot* 1988; **75**: 1099–113.

172. Smith SY and Stockey RA. Aroid seeds from the Middle Eocene Princeton Chert (*Keratosperma allenbyense*, Araceae): Comparisons with extant Lasioideae. *Int J Plant Sci* 2003; **164**: 239–50.

173. Nauheimer L, Metzler D and Renner SS. Global history of the ancient monocot family Araceae inferred with models accounting for past continental positions and previous ranges based on fossils. *New Phytol* 2012; **195**: 938–50.

174. Kvaček Z. *Limnobiophyllum* Krassilov—a fossil link between the Araceae and the Lemnaceae. *Aquat Bot* 1995; **50**: 49–61.

175. Stockey R, Hoffman G and Rothwell G. The fossil monocot *Limnobiophyllum scutatum*: Resolving the phylogeny of Lemnaceae. *Am J Bot* 1997; **84**: 355–68.

176. Bogner J. The free-floating aroids (Araceae)—living and fossil. *Zitteliana* 2009; **48/49**: 113–28.

177. Sille NP, Collinson ME, Kucera M *et al.* Morphological evolution of *Stratiotes* through the Paleogene in England: An example of microevolution in flowering plants. *Palaios* 2006; **21**: 272–88.

178. Chen LY, Chen JM, Gituru RW *et al.* Generic phylogeny, historical biogeography and character evolution of the cosmopolitan aquatic plant family Hydrocharitaceae. *BMC Evol Biol* 2012; **12**: 30.

179. Kar RK. The fossil floras of Kachchh IV. Tertiary palynostratigraphy. *Palaeobotanist* 1985; **34**: 1–280.

180. Harley MM. A summary of fossil records for Arecaceae. *Bot J Linn Soc* 2006; **151**: 39–67.

181. Dransfield J, Uhl NW, Asmussen CB *et al.* *Genera Palmarum: The Evolution and Classification of Palms*. London: Royal Botanic Gardens, Kew, 2008.

182. Ramírez SR, Gravendeel B, Singer RB *et al.* Dating the origin of the Orchidaceae from a fossil orchid with its pollinator. *Nature* 2007; **448**: 1042–5.

183. Guo YY, Luo YB, Liu ZJ *et al.* Evolution and biogeography of the slipper orchids: Eocene vicariance of the conduplicate genera in the Old and New World Tropics. *PLoS One* 2012; **7**: e38788.

184. Conran JG, Christophel DC and Cunningham L. An Eocene moncotyledon from Nelly Creek, Central Australia, with affinities to Hemerocallidaceae (Lilianae: Asparagales). *Alcheringa* 2003; **27**: 107–15.

185. Prasad V, Strömberg CA, Leaché AD *et al.* Late Cretaceous origin of the rice tribe provides evidence for early diversification in Poaceae. *Nat Commun* 2011; **2**: 480.

186. Christin P-A, Spriggs E, Osborne CP *et al.* Molecular dating, evolutionary rates, and the age of the grasses. *Syst Biol* 2014; **63**: 153–65.

187. Burke SV. Evolution of panic grasses (Panicoideae; Poaceae): A plastome phylogenomic study. *Doctoral thesis*. Northern Illinois University 2018.

188. Walther H. Ergänzung zur flora von Seifhennersdorf, Sachsen. *Abh Sta Mus Mineral Geol Dre* 1974; **21**: 143–85.

189. Walther H and Kvaček Z. Early Oligocene flora of Seifhennersdorf (Saxony). *Acta Mus Natl Pragae, Ser B Hist Nat* 2007; **63**: 85–145.

190. Grande L. Paleontology of the Green River Formation, with a review of the fish fauna, second edition. *Geol Sur Wyoming Bull* 1984; **63**: 1–333.

191. Osozawa S, Nackejima C and Wakabayashi J. Post-Triassic Spermatophyta timetree adding the Quaternary radiated *Asarum* wild gingers. *Res Sq* (10.21203/rs.3.rs-99466/v1).

192. Magallón S and Sanderson MJ. Absolute diversification rates in angiosperm clades. *Evolution* 2001; **55**: 1762–80.

193. Chandler MEJ. *The Lower Tertiary Floras of Southern England IV: A Summary and Survey of Findings in Light of Recent Botanical Observations*. London: British Museum of Natural History, 1964.

194. Mai DH. Entwicklung der Wasser- und Sumpfplanzen-Gesellschaften Europas von der Kreide bis ins Quartär. *Flora* 1985; **176**: 449–511.

195. Dorofeev PI. *Tertiary Floras in Western Siberia*. Leningrad: Akademia Nauk SSSR, Botanicheskii Institut VL Komarov, 1963.

196. Gregor HJ. *Schisandra geissertii* nova spec.—ein exotisches element im Elsäßer Pliozän (Sessenheim, Brunssumien). *Mitt bad Landesver Naturk Naturschutz* 1981; **12**: 241–7.

197. Denk T and Oh IC. Phylogeny of Schisandraceae based on morphological data: Evidence from modern plants and the fossil record. *Plant Syst Evol* 2005; **256**: 113–45.

198. Fan JH, Thien LB and Luo YB. Pollination systems, biogeography, and divergence times of three allopatric species of *Schisandra* in North America, China, and Japan. *J Syst Evol* 2011; **49**: 330–8.

199. Friis EM, Pedersen KR and Crane PR. Cretaceous diversification of angiosperms in the western part of the Iberian Peninsula. *Rev Palaeobot Palyno* 2010; **162**: 341–61.

200. Boucher FC, Zimmermann NE and Conti E. Allopatric speciation with little niche divergence is common among alpine Primulaceae. *J Biogeogr* 2016; **43**: 591–602.

201. Crawley M. Angiosperm woods from British Lower Cretaceous and Palaeogene deposits. *Spec Pap Palaeontol* 2001; **66**: 1–100.

202. Zanne AE, Tank DC, Cornwell WK *et al.* Three keys to the radiation of angiosperms into freezing environments. *Nature* 2014; **506**: 89–92.

203. Lott TA, Manchester SR and Dilcher DL. A unique and complete polemoniaceous plant from the Middle Eocene of Utah, USA. *Rev Palaeobot Palyno* 1998; **104**: 39–49.

204. Wheeler EF, Lee M and Matten LC. Dicotyledonous woods from the Upper Cretaceous of southern Illinois. *Bot J Linn Soc* 1987; **95**: 77–100.

205. Weyland H. Beiträge zur Kenntnis der Rheinischen Tertiärflora II. Erste Ergängzungen und Berichtigungen zur flora der Blätterkohle und des Polierschiefers von Rott im Siebengebirge. *Palaeontogr Abt B* 1937; **83**: 67–119.

206. Sims HJ, Herendeen PS, Lupia R *et al.* Fossil flowers with Normapolles pollen from the Upper Cretaceous of southeastern North America. *Rev Palaeobot Palyno* 1999; **106**: 131–51.

207. Crepet WL and Daghlian CP. Euphorbioid inflorescences from the Middle Eocene Claiborne formation. *Am J Bot* 1982; **69**: 258–66.

208. Gandolfo MA, Nixon KC and Crepet WL. Triuridaceae fossil flowers from the Upper Cretaceous of New Jersey. *Am J Bot* 2002; **89**: 1940–57.

209. Hertweck KL, Kinney MS, Stuart SA *et al.* Phylogenetics, divergence times and diversification from three genomic partitions in monocots. *Bot J Linn Soc* 2015; **178**: 375–93.

210. Tidwell WD and Parker LR. *Protoyucca shadishii* gen. et sp. nov., an arborescent monocotyledon with secondary growth from the middle Miocene of northwestern Nevada, U.S.A. *Rev Palaeobot Palyno* 1990; **62**: 79–95.

211. McKain MR, McNeal JR, Kellar PR *et al.* Timing of rapid diversification and convergent origins of active pollination within Agavoideae (Asparagaceae). *Am J Bot* 2016; **103**: 1717–29.

212. Ding L, Xu Q, Yue YH *et al.* The Andean-type Gangdese Mountains: Paleoelevation record from the Paleocene–Eocene Linzhou Basin. *Earth Planet Sci Lett* 2014; **392**: 250–64.

213. DeCelles PG, Quade J, Kapp P *et al.* High and dry in central Tibet during the Late Oligocene. *Earth Planet Sci Lett* 2007; **253**: 389–401.

214. Spicer R, Harris NBW, Widdowson M *et al.* Constant elevation of southern Tibet over the past 15 million years. *Nature* 2003; **421**: 622–4.

215. Hoke GD, Jing LZ, Hren MT *et al.* Stable isotopes reveal high southeast Tibetan Plateau margin since the Paleogene. *Earth Planet Sci Lett* 2014; **394**: 270–8.

216. Su T, Spicer RA, Li SH *et al.* Uplift, climate and biotic changes at the Eocene–Oligocene transition in south-eastern Tibet. *Natl Sci Rev* 2018; **6**: 495–504.

217. Ding L, Spicer RA, Yang J *et al.* Quantifying the rise of the Himalaya orogen and implications for the South Asian monsoon. *Geology* 2017; **45**: 215–8.

218. Gébelin A, Mulch A, Teyssier C *et al.* The Miocene elevation of Mount Everest. *Geology* 2013; **41**: 799–802.

219. Bird P. Formation of the Rocky Mountains, western United States: A continuum computer model. *Science* 1988; **239**: 1501–7.

220. Mix HT, Mulch A, Kent-Corson ML *et al.* Cenozoic migration of topography in the North American Cordillera. *Geology* 2011; **39**: 87–90.

221. Sahagian D, Proussevitch A and Carlson W. Timing of Colorado Plateau uplift: Initial constraints from vesicular basalt-derived paleoelevations. *Geology* 2002; **30**: 807–10.
